# Adaptive Laboratory Evolution in *Synechocystis* sp. PCC 6803: Current Status and Perspectives

**DOI:** 10.3390/microorganisms14081836

**Published:** 2026-08-19

**Authors:** Dielle P. Procópio, Anna Santin, Cassius V. Stevani

**Affiliations:** 1 Departamento de Química Fundamental, Instituto de Química, Universidade de São Paulo, São Paulo 05508-000, Brazil; 2 Dipartimento di Biologia, Università Degli Studi di Padova, 35121 Padova, Italy; 3 Departamento de Bioquímica, Instituto de Química, Universidade de São Paulo, São Paulo 05508-000, Brazil

**Keywords:** *Synechocystis* sp. PCC 6803, adaptive laboratory evolution, cyanobacteria, microbial evolution, stress tolerance

## Abstract

With the increasing environmental concerns about carbon dioxide emissions and the pressing demand for sustainable resources, photosynthetic microorganisms have gained considerable attention as alternative platforms for the environmentally friendly production of fuels and chemicals. These organisms function as green cell factories capable of directly converting carbon dioxide into organic carbon metabolites using solar energy, offering a promising platform for more sustainable biomanufacturing. Among these organisms, cyanobacteria, and particularly *Synechocystis* sp. PCC 6803, have emerged as particularly attractive hosts due to their relatively simple cellular organization, efficient photosynthetic metabolism, and amenability to genetic manipulation. In addition to rational metabolic engineering approaches, Adaptive Laboratory Evolution (ALE) has recently been proposed as a powerful strategy to improve *Synechocystis* strain robustness, enhance tolerance to environmental and metabolic stresses, and optimize cellular performance under specific growth conditions. By selecting beneficial spontaneous mutations over successive generations, ALE could complement genetic engineering strategies and further expand the potential of cyanobacterial platforms for efficient and sustainable bioproduction.

## 1. Introduction

Cyanobacteria are microbial cell factories capable of converting CO_2_ into valuable chemicals through photosynthesis. They can achieve relatively high growth rates and, unlike crops, do not require arable land for cultivation [1,2]. In particular, the well-characterized cyanobacterium *Synechocystis* sp. PCC 6803 (hereafter *Synechocystis*) is a versatile unicellular prokaryote with a unique combination of molecular, physiological, and morphological traits that have made it one of the most widely used model organisms. It can support diverse lifestyles, such as phototrophic, heterotrophic, or mixotrophic growth [3,4], and can tolerate a wide range of environmental stresses [5,6,7,8,9,10]. *Synechocystis* is widely recognized as a genetically tractable model organism due to its natural competence for genetic transformation, which facilitates studies of cyanobacterial metabolism and photosynthesis. The *Synechocystis* genome has been fully sequenced, and a wide range of molecular tools is available, including antibiotic resistance markers, well-characterized promoters, and genome editing tools [11,12].

**Figure 1:**
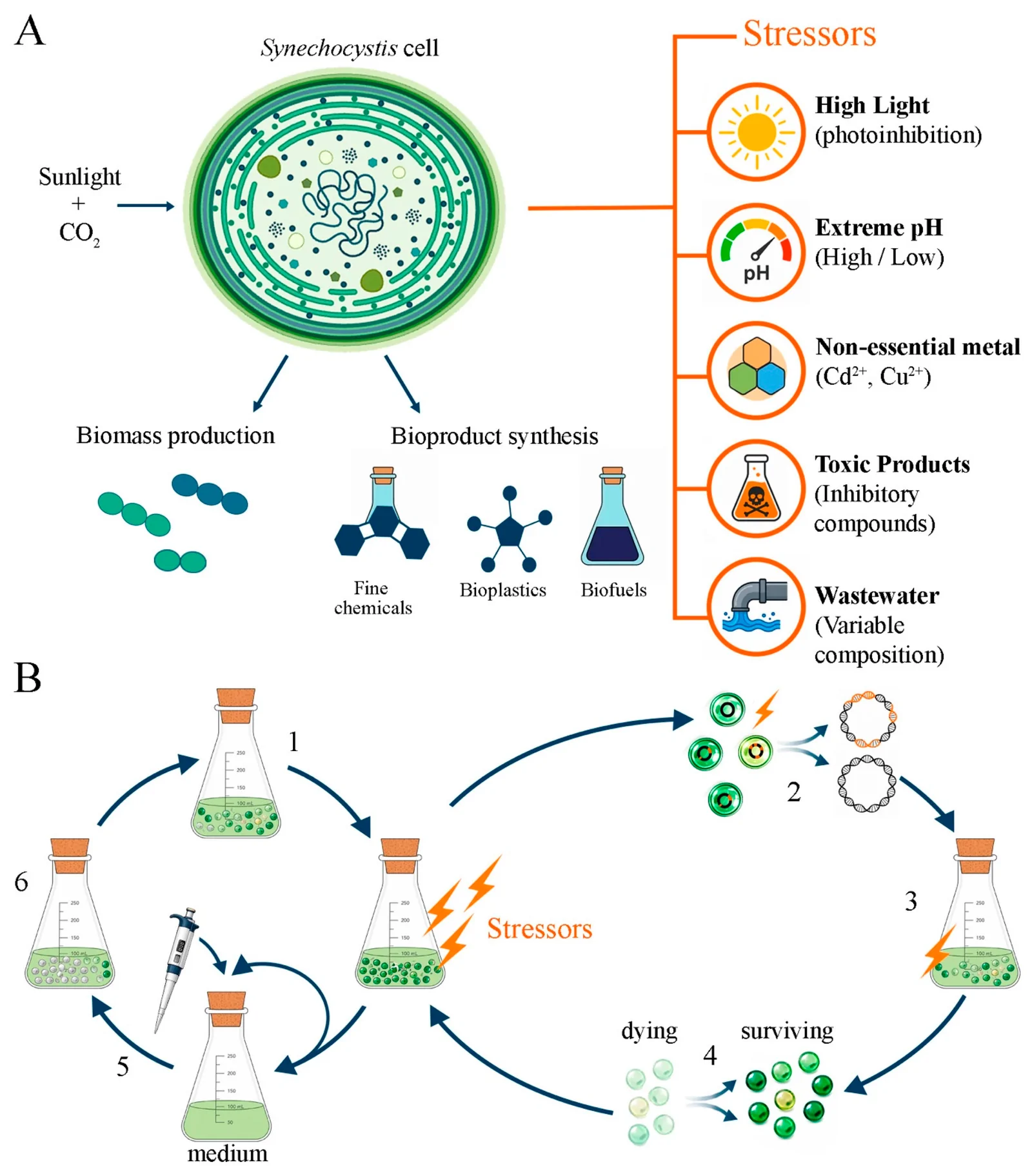
Overview of ALE applied to *Synechocystis*. (**A**) *Synechocystis* converts CO_2_ and sunlight via photosynthesis into biomass and diverse bioproducts, including fine chemicals, bioplastics, and biofuels. Industrial cultivation exposes the organism to multiple stressors, such as high light intensity, extreme pH, non-essential metals, toxic endogenous or heterologous compounds, and wastewater with variable composition, which constrain productivity and scalability. (**B**) Schematic representation of the ALE workflow: (1) populations are exposed to specific environmental stressors; (2) spontaneous mutations continuously arise within the population, generating phenotypic heterogeneity upon which the imposed selective pressure acts; (3) continued exposure leads to the proliferation of fitness-enhancing variants; (4) natural selection occurs as sensitive cells perish while robust mutants survive; (5) adapted populations may be periodically transferred into fresh medium to maintain selection and cumulative adaptation; (6) after successive cycles, evolved strains emerge with enhanced tolerance and improved performance under selected conditions.

Despite these advances, the industrial implementation of cyanobacteria and their products is currently constrained by several limitations. First, product yields often remain insufficient for economic competitiveness, requiring metabolic optimization. Second, the inherent tolerance of cyanobacteria to both their own products (endogenous compounds such as amino acids or secondary metabolites) and externally supplied chemicals (e.g., biofuels or solvents) is often low, limiting the achievable titers during production [13]. Third, industrial cultivation conditions often impose environmental stresses that impair growth and productivity. For example, outdoor or wastewater-based media expose cells to challenges such as high light intensity, pH extremes, and non-essential metal exposure, requiring enhanced stress tolerance (Figure 1A) [14,15,16].

Addressing these limitations is essential to enhance the feasibility and scalability of cyanobacterial bioprocesses. Several approaches have been explored to tackle these challenges and thus increase tolerance to both environmental stresses and internal products. Targeted mutagenesis and metabolic engineering are often employed to enhance product yields by rationally modifying specific known pathways [2,17,18]. Improving stress or product tolerance remains challenging because these traits are typically polygenic, arising from the coordinated effects of multiple genes rather than a single genetic determinant, which substantially complicates genome editing efforts [13,14,19]. In this context, strategies such as random mutagenesis or Adaptive Laboratory Evolution (ALE) have demonstrated great promise [20,21]. ALE applies sustained selective pressure to enrich populations for desirable phenotypes, allowing cells to gradually adapt to environmental or chemical stresses while retaining overall fitness (Figure 1B). This approach is especially useful for tolerance traits, which are typically complex, multifactorial, and difficult to engineer rationally [21,22].

ALE has been successfully applied to cyanobacteria, including *Synechocystis*, to enhance tolerance to environmental stresses, including high light, acidic pH, or non-essential metals. These conditions are generally relevant to outdoor cultivation or wastewater-based production, where enhanced tolerance may improve strain performance and, when coupled to appropriate production pathways, potentially support productivity. ALE has also been employed to increase resistance to products that are often toxic to cyanobacteria, including endogenously produced compounds resulting from pathway overexpression (e.g., amino acids or other metabolites) or heterologously produced compounds via the introduction of new biosynthetic pathways (e.g., biofuels). By leveraging ALE, researchers can systematically evolve *Synechocystis* strains with superior tolerance, ultimately addressing key limitations in robustness and scalability while maintaining the benefits of cyanobacterial systems for sustainable biotechnology.

## 2. Literature SEARCH and Study Selection

This study consists of a narrative review that does not follow a formal systematic review protocol. Relevant studies were identified through literature searches in Google Scholar and PubMed (last conducted on 10 August 2026), using combinations of the keywords “adaptive laboratory evolution”, “ALE”, “adaptive evolution”, “experimental evolution”, “evolutionary engineering”, “tolerance evolution”, “serial passaging”, “long-term selection” and “genome resequencing”, each combined with “*Synechocystis*” or “*Synechocystis* sp. PCC 6803”. Twenty-one search queries were tested (10 in Google Scholar and 11 in PubMed). The search results were screened within the records accessible through the databases; in Google Scholar, the number of records that could be screened was limited by the database’s pagination restrictions. Across the complete search process, 288 accessible records were consolidated, of which 10 duplicate records were removed, leaving 278 unique records for screening by title and abstract. Of these, 259 records were excluded (55 did not perform ALE; 86 were review or perspective articles; 69 were not peer-reviewed original research, including preprints, theses, book chapters, or conference abstracts; and 49 did not use *Synechocystis* sp. PCC 6803). No publication date restriction was applied, and only peer-reviewed articles published in English were considered. The search process identified 19 eligible studies, which are included in Table 1. The complete search process is summarized in Supplementary Figure S1, and the full search log, including the search queries, retrieval results, screening decisions, and reasons for exclusion, is provided in Supplementary Table S1.

Original research articles reporting adaptive laboratory evolution experiments in *Synechocystis* were included when a population was subjected to a defined and sustained selective pressure over serial or continuous passage, allowing naturally arising or experimentally introduced genetic variation to be selected over time. Studies focused exclusively on physiological acclimation, directed evolution of isolated enzymes or proteins, mutagenesis without subsequent serial adaptive selection, long-term maintenance or passage without a directional selective pressure, review or perspective articles, and studies not involving *Synechocystis* were excluded. Studies combining mutagenesis with subsequent serial or continuous selection were included and classified as “mutagenesis-assisted ALE” when long-term adaptive selection, rather than mutagenesis alone, was responsible for the evolved phenotype.

## 3. ALE: Principles and Mechanisms

**Figure 2:**
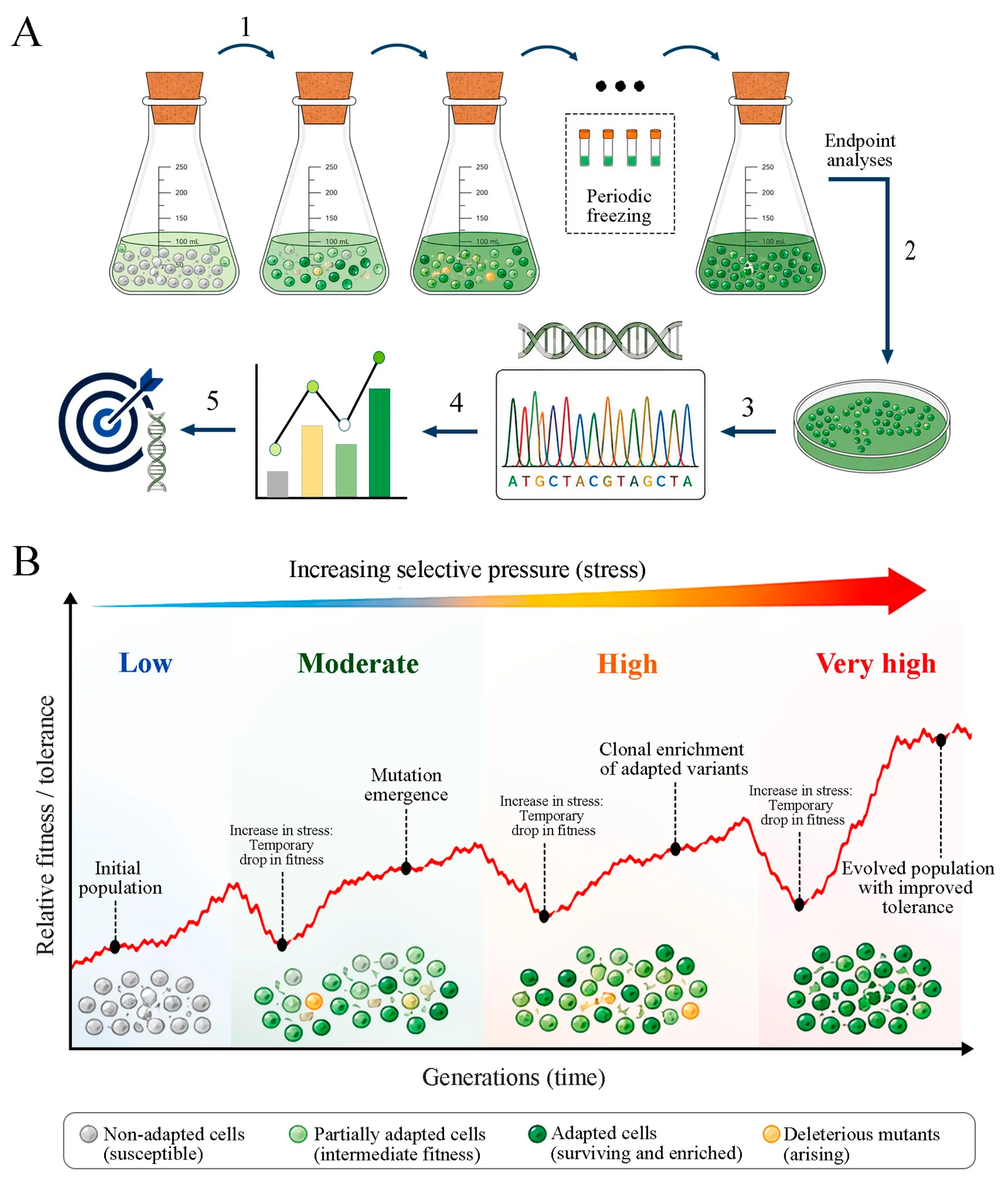
Experimental framework and evolutionary dynamics of ALE. (**A**) Schematic overview of a standard ALE workflow. (1) Serial passaging of microbial populations under selective pressure. Transfers are performed according to predefined rules (e.g., inoculum size, transfer interval, and growth phase). The selective pressure may remain constant or be progressively increased depending on the experimental design. Evolving populations are periodically cryopreserved to capture intermediate evolutionary states. Following the evolution experiment, endpoint analyses are performed, including (2) isolation of evolved colonies on solid medium, (3) whole-genome sequencing of evolved clonal isolates, (4) phenotypic characterization and identification of superior evolved strains, and (5) causal reconstruction and validation of candidate adaptive mutations through targeted genetic reconstruction and phenotypic confirmation. (**B**) Conceptual representation of the adaptive dynamics during ALE. The red curve illustrates changes in relative fitness under either constant or progressively increasing selective pressure over successive generations. Following each increase in selective pressure, the population may experience a temporary reduction in relative fitness before recovering through the selection and enrichment of beneficial variants. As adaptation proceeds, partially adapted populations become progressively enriched until an evolved population with improved tolerance to the imposed selective condition is obtained. Cell colors indicate non-adapted (susceptible), partially adapted, adapted, and deleterious mutant subpopulations.

ALE is commonly performed through serial passaging of microbial populations under selective pressure, such as stepwise increases in light, substrate concentration, osmotic stress, or inhibitor levels (Figure 1B and Figure 2A). Cultures are transferred repeatedly into fresh medium containing incrementally higher stress levels once stable growth is achieved at the previous stress level. This process is carried out over hundreds of generations, allowing spontaneous mutations to arise and be enriched through natural selection [16,23,24]. Through continuous serial passaging, spontaneous genetic variants with improved growth, tolerance, or metabolic performance are naturally selected (Figure 2A,B), yielding evolved strains with desirable phenotypic traits without requiring prior knowledge of the underlying genetic mechanisms [14,21].

The success of ALE depends on four fundamentally distinct evolutionary processes that shape the emergence and fixation of adaptive mutations. First, mutations arise randomly and independently of the selective environment (mutation supply). The imposed condition then determines which of the resulting variants survive and reproduce (selection). During serial passaging, population bottlenecks amplify the effects of genetic drift, leading to stochastic losses of variants irrespective of their fitness. Finally, in large asexual populations, multiple beneficial lineages often arise independently and compete for fixation before any lineage reaches fixation (clonal interference) [25,26].

ALE can be carried out using various approaches, including colony transfer, serial liquid transfer via batch cultivation in shake flasks, and chemostat cultures. Colony transfer involves repeatedly selecting single colonies on agar plates, which is particularly useful for organisms that form aggregates or when liquid serial transfer is not applicable [27]. Serial liquid transfer is the most widely used approach because of its simplicity and speed. This method involves the periodic dilution of a batch culture into fresh medium once the desired growth phase is reached [21]. Serial liquid transfer subjects the population to fluctuating nutrient concentrations and alters growth phases, which can drive adaptations to both the stressor and the feast-and-famine cycles. The use of a chemostat culture for ALE offers a more controlled alternative. Although the chemostat culture method requires more complex cultivation systems than colony transfer and serial transfer methods, it is less labor-intensive once the operation begins [14]. Chemostat culture results in a greater number of cell divisions and thereby generates greater genetic diversity, which may ultimately improve the efficiency of ALE [23,24,28].

Beyond the cultivation format, the most critical feature of the experimental design is the definition of the selection strategy. For *Synechocystis*, two main selection strategies are commonly used: constant selection, where a fixed high-stress level is maintained, and stepwise selection, which progressively increases the selective challenge. Stepwise selection can reduce the risk of population extinction while progressively increasing the selective challenge and may allow sequential enrichment of variants with different fitness effects [14]. This dynamic design allows for the accumulation of multiple small-effect mutations that collectively provide high tolerance. In polyploid cyanobacteria, prolonged selection may also facilitate the segregation and enrichment of adaptive alleles across chromosome copies, although complete segregation must be experimentally verified.

To identify the molecular basis of evolved phenotypes, whole-genome sequencing of evolved strains relative to their parental background has become the primary method for identifying adaptive mutations, driven by the increasing availability of high-throughput, low-cost sequencing technologies (Figure 2A) [19,29]. Whole-genome sequencing identifies the complete set of mutations acquired during the experiment, including single-nucleotide polymorphisms (SNPs), insertions, deletions, and copy number variants; moreover, when combined with population-level sequencing across time points, whole-genome sequencing can reveal the temporal dynamics of mutation fixation and the dynamics of adaptive evolution [14]. Transcriptomic analysis complements this by revealing which regulatory pathways are differentially activated in the evolved strain, providing a mechanistic context for how genomic changes translate into altered gene expression and metabolic phenotype [14]. Proteomic and metabolomic profiling extend this analysis to the level of protein abundance and metabolic flux, respectively, allowing the detection of adaptive remodeling in pathways that do not show obvious genomic signatures [19].

## 4. Positioning ALE Within the Mutational Landscape

ALE can be used either as an alternative to other mutational approaches or in combination with them because of its versatility. One of its major advantages is that selection acts on spontaneously arising mutations across the entire genome, rather than from a pre-specified set of candidate loci. While rational metabolic engineering relies on the a priori identification of specific genetic targets and mechanistic hypotheses, ALE operates without prior assumptions about the genetic architecture of the desired phenotype [14,19,21]. This feature is particularly advantageous for complex traits such as stress tolerance, which do not arise from single-pathway perturbations but rather from the emergent interactions of transcriptional networks, membrane remodeling, and global metabolic flux distributions [14,21,30]. Consequently, rational strategies are most straightforward to apply when the genotype-to-phenotype relationship is well characterized and oligogenic. When the relationship is diffuse, polygenic, and condition-dependent, iterative rational approaches become considerably more resource-intensive because each design cycle still depends on prior mechanistic hypotheses. ALE does not require this dependency, as selection acts directly on spontaneously arising variation, allowing mutations in global regulatory systems that would not have been chosen a priori to emerge through selection and reveal non-intuitive targets for subsequent validation [31]. Accordingly, ALE should be viewed as complementary to, rather than a replacement for, rational engineering approaches [32].

Importantly, ALE and rational engineering are not mutually exclusive. Rather, they function most powerfully as complementary phases within an iterative design–build–test–learn framework [14,33]. ALE-derived mutations serve as high-confidence hypothesis-generating tools, as the genetic variants enriched through natural selection define prioritized targets for subsequent rational reconstruction, causal validation, and combinatorial engineering. Conversely, rationally engineered strains carrying specific pathway modifications can serve as evolved starting points for ALE, concentrating the evolutionary search within a more constrained and favorable fitness landscape [19,32].

Compared with other untargeted approaches such as random chemical or physical mutagenesis (e.g., treatment with alkylating agents, UV irradiation) and insertional mutagenesis, ALE offers a fundamentally distinct mutational regime that shapes both the quantity and the functional relevance of the mutations recovered. Random mutagenesis approaches generate large numbers of mutations throughout the genome, enabling rapid identification of mutants with strong, measurable phenotypic effects, particularly those arising from single loss-of-function events [21]. However, because mutations are introduced non-selectively and at high rates, the resulting strains typically carry a substantial number of neutral or deleterious mutations alongside any beneficial ones, imposing a genetic load that can compromise strain stability, reproducibility, and industrial applicability [14,26]. Random mutations are rarely beneficial; the vast majority are neutral or mildly deleterious, and an increase in mutation rate beyond a certain threshold accelerates the accumulation of this genetic load rather than adaptive improvement [26,31]. ALE circumvents this limitation by coupling the natural, low-rate supply of spontaneous mutations with continuous, iterative selection. Rather than generating diversity and screening in separate steps, ALE allows natural selection to act on spontaneously arising mutations in real time, thereby continuously enriching variants with genuine fitness advantages under the imposed condition. This selective filter enables ALE to identify mutations with individually small phenotypic effects that would be undetectable in a single-round mutagenesis and screening strategy. However, multi-round mutagenesis protocols coupled with iterative selection can likewise enrich small-effect variants [14,21].

Under single-round screening, a mutant carrying a small-effect beneficial mutation remains indistinguishable from the wild-type population within the experimental noise and is discarded. Under continuous selective filtering, the same mutant is progressively enriched across generations, becoming detectable and isolable. This temporal amplification allows ALE to identify cumulative small-effect mutations underlying complex polygenic phenotypes such as stress tolerance, provided selection is sustained over enough generations. Other iterative selection strategies can likewise enrich such variants under appropriate experimental designs.

Insertional mutagenesis occupies an intermediate position in this landscape. It enables systematic, genome-wide loss-of-function screening with single-gene resolution and has been used productively to identify tolerance-associated loci [34,35]. However, insertional mutagenesis is structurally limited to the identification of genes whose disruption is beneficial. This constraint excludes gain-of-function mutations and regulatory rewiring, although it can still recover small-effect loss-of-function alleles given sufficient screening depth. Collectively, these distinctions position ALE not as a replacement for mutagenesis-based approaches but as a complementary strategy for navigating complex, polygenic fitness landscapes, particularly when selection can be sustained over many generations [14].

ALE also has intrinsic limitations that should be considered during experimental design and strain evaluation. First, ALE is not universally applicable as its implementation requires that the desired phenotype be directly coupled to a measurable growth or survival advantage under the imposed laboratory condition. When the target phenotype does not confer a direct growth or survival advantage (i.e., is fitness-neutral), natural selection will systematically enrich non-producing variants that grow faster, working against the engineering objective. In such cases, prior metabolic engineering strategies must be employed to artificially couple the desired phenotype to cellular growth before ALE can proceed effectively [14,36]. Second, fitness gains achieved under the selective condition may be accompanied by trade-offs in other phenotypic properties; neutral or mildly deleterious mutations can be carried alongside adaptive ones, and the transferability of evolved phenotypes across conditions is not guaranteed [14]. These limitations underscore the need for systematic post-ALE characterization, including whole-genome resequencing, causal validation of candidate mutations, and phenotypic assessment under conditions relevant to the intended industrial application, not only under the selective condition used during evolution.

The duration of ALE experiments is also a critical and often underappreciated parameter. Experimental duration is typically expressed in generations or, more precisely, in cumulative cell divisions. This metric accounts for population bottlenecks during serial passaging and is more directly proportional to the probability of mutation occurrence [37]. While most ALE studies span 100 to 2000 generations, long-term evolution experiments suggest that fitness gains can continue indefinitely, with *Escherichia coli* populations having been propagated for over 75,000 generations in the landmark long-term evolution experiment initiated by Lenski and colleagues [38,39]. Determining when to terminate an ALE experiment based on a fixed generation count remains inherently arbitrary. Evolved strains are considered experimental endpoints by convention, not because adaptation has ceased. For practical biotechnology applications, it is therefore essential to balance phenotypic improvement with resource efficiency, and to recognize that the optimal evolved strain for industrial deployment is not necessarily the one with the highest fitness under the selective condition, but the one that best combines performance gains with stability and minimal trade-offs under process-relevant conditions [21].

## 5. ALE Applications in *Synechocystis*

ALE has proven particularly effective for enhancing tolerance to high substrate or product concentrations. Whether this translates into increased product yield or productivity depends on the subsequent incorporation of the tolerant background into an engineered production pathway, which has been directly tested in only a subset of the *Synechocystis* studies reviewed here [14,40,41]. To date, many microorganisms, including *E. coli*, *Halomonas bluephagenesis,* and *Saccharomyces cerevisiae,* have been successfully subjected to ALE to produce desired bioproducts using acetate as the carbon source, such as ethanol, polyhydroxybutyrate (PHB), recombinant protein, and 3-hydroxypropionic acid [14,24,42,43,44]. Nevertheless, the maturity and throughput of ALE differ substantially across microbial systems, and this disparity is directly relevant to the challenges of applying ALE to *Synechocystis*.

*Synechocystis* presents a distinct set of biological constraints that fundamentally shape the design, duration, and interpretation of ALE experiments. Its photoautotrophic lifestyle introduces light as an additional and variable parameter that must be controlled throughout the experiment to ensure that phenotypic changes reflect adaptation to the intended selective pressure rather than to fluctuations in energy supply [45]. Its generation time of approximately 6 to 24 h under standard conditions means that reaching generation numbers comparable to those achieved in *E. coli* or *S. cerevisiae* requires substantially longer calendar time, with ALE experiments in *Synechocystis* spanning from several weeks to more than two years. Furthermore, *Synechocystis* is highly polyploid, which is a feature that simultaneously buffers newly arising mutations from immediate exposure to selection conditions and prolongs the time required for beneficial alleles to reach fixation across all genome copies [46,47].

Many ALE experiments in *Synechocystis* have used repeated serial transfers of the culture into fresh medium containing the selective agent at sublethal concentrations (Table 1).

**Table 1:** Overview of Adaptive Laboratory Evolution (ALE) applied to *Synechocystis* sp. PCC 6803. The table summarizes the evolved phenotypes, ALE strategies, cultivation conditions, and highest levels of evidence supporting the adaptive role of the identified mutations. The evidence levels are classified as candidate association, replicated convergence, and causal validation; the levels were validated in an engineered producer or process-relevant condition according to the highest level of reverse-genetic validation performed in each study.

Characteristics of the Evolved Strain	ALE Process/Strategy	Standardized Culture Conditions	Evidence/Functional Validation	Reference
Environmental stressors				
High light tolerance	Mutagenesis-assisted ALE: serial passaging of populations into a condition that combines mutagenesis by exposure to methyl methane sulfonate or UV light in different permutations, and then exposure to increasing light intensities, with an initial OD_730_ of 0.1 and ending at OD_730_ of 2.0	23 °C, 30 µmol photons m^−2^s^−1^ (routine maintenance) ALE selection ramped up to 2300 µmol photons m^−2^s^−1^; BG_11_ medium	Causal validation: *ndhF1* and *fusB* individually reconstructed and confirmed causal	[48]
High light tolerance	Serial passaging of populations in fresh medium every 2–3 days for 52 days under high-light selection at 7000–9000 µmol photons m^−2^s^−1^	34 °C, 40 µmol photons m^−2^s^−1^ (routine maintenance; ALE selection at 7000–9000 µmol photons m^−2^s^−1^); modified BG_11_ medium aerated with sterile air	Causal validation: *hik26* and *slr1916* validated by knockout and complementation	[49]
Fluctuating-light tolerance	Serial passaging of 6 independent populations for 20 selection cycles over 20 months under two progressively intensified fluctuating-light regimes (non-lethal and lethal to the ancestor), with increasing high light intensity (700–1200 µmol photons m^−2^s^−1^) and, in the lethal regime, progressively shorter low-light intervals	23 °C; fluctuating-light regimes intensified from 700/50 to 1200/12 µmol photons m^−2^s^−1^; BG_11_ medium	Causal validation: *sll0518*, *pam68*, and *rpaB-T183P* individually reconstructed; convergent *rpaB-D194G* not tested	[45]
Salt tolerance	Serial passaging of 10 independent populations every 7 days at an initial OD_730_ of 0.2; 303 days of effective exposure to 3% NaCl (~43 cycles)	30 °C, 30 ± 5 µmol photons m^−2^s^−1^; BG_11_ supplemented with 3% NaCl	No genomic or causal validation; metabolomic/transcriptomic analyses identified divergent responses among evolved strains	[50]
Salt tolerance	Serial passaging of a population under progressively increasing NaCl concentrations (4.0–6.5% NaCl), with 0.1% NaCl increments following adaptation at each level (~125 passages, 856 days)	30 °C, 50 µmol photons m^−2^s^−1^; BG_11_ supplemented with 4.0–6.5% NaCl	Causal validation: *slr1670* deletion and evolved *slr1753* overexpression enhanced salt tolerance; the combined modification impaired growth, suggesting a negative genetic interaction	[51]
Cadmium tolerance	Serial passaging of a single population in fresh medium for 128 passages (802 days), with the concentration raised once growth reached an OD_750_ of 0.5 (from an initial 0.1) within 96 h	30 °C, 50 µmol photons m^−2^s^−1^; BG_11_ supplemented with varying concentrations of cadmium (CdSO_4_), whose concentration was gradually increased from 4.6 µM to 9.0 µM	Causal validation: *slr0454*, *slr0623*, *slr0721*, and *slr0798* validated via overexpression (the evolved/truncated *slr0454* allele showing the clearest single causal effect); *slr0774* and *slr1753* supported only by knockout; *slr1302*, *ssr1480*, and *sll1586* not confirmed	[52]
Cadmium and high light tolerance	Serial passaging of three parallel lineages derived from the high-light-adapted parent strain, under stepwise increasing Cd^2+^ concentrations (3.0–5.5 mM) for ~800 days; 3 evolved isolates obtained (one per lineage)	30 °C, 600 µmol photons m^−2^s^−1^. BG11 medium supplemented with CdSO_4_	Causal validation: 4 of 15 mutated loci (*ssl2615*, *sll1732*, *ssr1480*, and *sll1659*) identified by knockout and complementation	[53]
Acid tolerance	Serial passaging of 2 independent populations in fresh medium every 7 days for 3 months, kept below OD_730_ of 0.05 at each dilution	30 °C, 30 µmol photons m^−2^s^−1^. 3% CO_2_-enriched aeration; BG_11_ medium buffered with MES-NaOH, whose pH was reduced gradually from 6.0 to 5.5	Replicated convergence: ATP synthase operon repeatedly targeted; no causal reconstruction	[54]
High temperature tolerance	Mutagenesis-assisted ALE: random mutagenesis (2× UV and 2× MMS) followed by in vivo selection under increasing temperature, generating a thermotolerant strain mixture (mutIV-mix)	Temperature and light progressively increased from 30 °C/~3 µmol photons m^−2^ s^−1^ to 47 °C/~85 µmol photons m^−2^ s^−1^ during the day; night at 26 °C in the automated screening system; modified BG11 (mBG11) supplemented with Instant Ocean^®^	Replicated convergence: recurrent mutations in *clpC* and *pnp*; no causal reconstruction	[55]
High temperature and high light tolerance	Serial passaging of 3 parallel lineages derived from a previously high-light-tolerant strain (not a wild-type strain), under constant high light intensity (750 µmol photons m^−2^ s^−1^) with progressively increasing cultivation temperatures from 35 to 40 °C; 81 passages over 700 days	35–40 °C, constant 750 µmol photons m^−2^ s^−1^; BG_11_ medium	Causal validation: *slr1329* (AtpB) showed the strongest causal evidence through gene inhibition, overexpression, and in situ replacement in HL-4; *sll1457* showed a weaker positive effect in HL-4 after heat shock, whereas *sll1626* did not show a clear advantage in the in situ replacement experiment	[56]
Copper and moxifloxacin tolerance	Chronic exposure of 3 independent replicate populations to Cu^2+^ (10, 50, and 250 µg L^−1^) or moxifloxacin (0.2, 2, and 20 µg L^−1^) for 140 days (~20 weekly passages); subsequent analyses focused on the highest concentrations (250 µg L^−1^ Cu^2+^ and 20 µg L^−1^ moxifloxacin)	28 ± 1 °C; continuous illumination (2000 lx); BG_11_ medium supplemented with Cu^2+^ and/or moxifloxacin	Candidate association: multi-omics associations, including *rpoB* mutations; no knockout, complementation, or allele-replacement validation; stability test distinguished stable tolerance to the primary stressors from reversible cross-tolerance	[57]
Triclosan tolerance	ALE arm of a hypermutation-comparison study: serial passaging of triplicate 100 mL cultures under constant triclosan (0.8 mg L^−1^) for 32 cycles (162 days)	27 ± 1 °C; 50 µmol photons m^−2^s^−1^; BG_11_ medium supplemented with triclosan	Causal validation: *fabI* (A116V), detected in all TCS-tolerant strains, was reconstructed in WT and conferred enhanced TCS tolerance; molecular docking predicted reduced TCS binding affinity	[58]
Adaptation to wastewater cultivation	Repeated cycles of cultivation in synthetic wastewater (MBG), followed by single-colony isolation and expansion in BG11, over 374 days of cumulative selection	30 °C, light intensity and photoperiod were not specified for the ALE regimen; synthetic wastewater (MBG medium)	Candidate association: RNA-seq and photosynthetic-oxygen-evolution evidence only; no whole-genome resequencing or causal validation	[59]
Tolerance to products				
Phenylalanine	Two-stage selection: spontaneous resistant colonies first isolated on BG11 agar with 1 mM phenylalanine (5–7 days), followed by serial liquid subculturing with stepwise increasing phenylalanine concentration until stable growth at 12 mM	30 °C, 45 µmol photons m^−2^ s^−1^ for routine cultivation; selection/escalation phase specifically described as “low light intensity”, value not given; BG_11_ medium supplemented with phenylalanine	Replicated convergence: all ten isolates mutated *ccmA*; no causal reconstruction	[60]
Toxic amino acid tolerance	Serial passaging of a single population for 85 transfers (408 days; 548 generations), initially under gradually increasing concentrations of six toxic amino acids and subsequently in TCM1	30 °C, 10 µmol photons m^−2^s^−1^; TCM_x_ medium containing vitamins, nucleosides, and glucose, with gradually increasing concentrations of six toxic amino acids; after day 79, TCM1 was used for continued selection	Candidate association: population sequencing only; no causal validation	[61]
n-Butanol tolerance	Serial passaging of four independent *Synechocystis* populations for 94 passages (395 days; ~700 generations), with cultures transferred when they reached the middle exponential phase (OD_730_ of 0.5)	30 °C, 50 µmol photons m^−2^ s^−1^, 130 rpm; BG_11_ medium supplemented with varying concentrations of butanol, with an initial concentration of 0.2% *v*/*v*, which was gradually increased to 0.5% *v*/*v*	Candidate association: metabolomic evidence only; no whole-genome sequencing or causal testing	[1]
Isobutanol tolerance	Serial passaging of four independent parallel cultures in fresh medium every 3.5 days at an initial OD_730_ of 0.03 over approximately 1824 h of evolution	34 °C, 40 µmol photons m^−2^ s^−1^, 150 rpm; BG_11_ medium supplemented with 50 mM NaHCO_3_ and 2 g L^−1^ isobutanol	Validated in engineered producer: *mcpA* and *envD*: exact evolved mutations reconstructed; *hik43*: full deletion tested (not exact mutation); epistasis tested via double deletions; ethanol pathway introduced into evolved background demonstrated increased ethanol productivity of 142% of the control	[40]
Engineering + ALE				
PSI-independent photoautotrophic growth	Two ALE campaigns: (1) 12 parallel PSI-deficient lines recovering photoautotrophic growth under glucose-free, very low-light conditions for up to 19 weeks (8/12 succeeded); (2) confirmatory ALE of a Δ*psaAB fusA*^I28T^ strain using >100 replicate cultures under a gradual glucose-restriction regime, yielding 3 additional evolved lines after ~15–16 weeks	30 °C, 3–5 µmol photons m^−2^ s^−1^ (campaign 1 mixotrophic pre-culture at ~10 µmol photons m^−2^ s^−1^ with 1.25 mM glucose); BG11 medium, glucose-free during selection (campaign 2 used a stepwise glucose-restriction gradient from 0.90 mM down to 0.12 mM)	Causal validation: recurrent *fusA* alleles were reconstructed in the PSI-deficient parental backgrounds and restored photoautotrophy; *ndhB* was required, whereas *pgr5* was dispensable; in the Δ*psaAB* background, *fusA*^I28T^ alone was insufficient, indicating that additional adaptive mutation(s) were required	[62]
Enhanced mannitol production under salt stress	Serial passaging of a compatible-solute-deficient *Synechocystis* strain and two engineered mannitol-producing derivatives in three parallel cultures under either constant 200 mM NaCl or progressively increasing NaCl concentrations (200–400 mM)	30 °C, ~50 µmol photons m^−2^s^−1^ red light, 120 rpm; BG_11_ medium with 50 mM PIPPS buffer (pH 8), supplemented with NaCl	Validated in engineered producer: partial *pnp* deletion increased mannitol production ~6.5-fold, although it reduced growth and salt tolerance; complementation was not performed	[41]

For each study in Table 1, additional experimental design fields, including the ancestral substrain and resequencing status, selection regime, culture vessel and CO_2_ supply, transfer criteria, number of independent lineages, mutation types, sequencing at the population or clone level, reverse-engineering validation, stability without selection, cross-tolerance, and process-relevant trade-offs, are provided in Supplementary Tables S2 (experimental design and cultivation conditions) and S3 (genomic outcomes and functional validation).

### 5.1. Tolerance to Environmental Stressors

Improving tolerance to environmental stressors is essential for expanding the range of cultivation conditions suitable for *Synechocystis*, particularly in environments that are currently challenging for stable and efficient growth. Increased robustness is crucial to ensure reliable large-scale cultivation and to broaden the operational window of industrial processes. In this context, ALE provides an effective strategy for selecting strains with enhanced resilience, enabling more stable performance under cultivation conditions relevant for industrial-scale applications.

#### 5.1.1. High-Intensity Light Tolerance

Whether natural or anthropogenic, environmental fluctuations impose significant barriers to the industrial scalability of *Synechocystis*. Adaptation to extreme irradiance highlights how ALE can push photosynthetic limits. Intensities above 200 μmol photons m^−2^ s^−1^ typically trigger reactive oxygen species (ROS) formation and severe photodamage to cellular components, which is a phenomenon known as photoinhibition [63]. Evolved ALE *Synechocystis* populations have demonstrated a remarkable capacity to grow under extreme irradiances, reaching 2300 μmol photons m^−2^ s^−1^ [48] or even 9000 μmol photons m^−2^ s^−1^ [49].

This robust phototolerance was achieved through a reconfiguration of the photosynthetic apparatus, maintaining chlorophyll levels and the integrity of thylakoid protein complexes [48], which supported growth under irradiance levels that inhibited the wild-type strain. Genomic analysis of these independently evolved populations revealed a complex mutational landscape. Dann et al. reported that 196 mutant alleles were identified exclusively in the high-intensity light-adapted strains, comprising 108 non-synonymous SNPs and various structural variations, including deletions and insertions. Several alleles reached high frequency (≥80%) in independently evolved lineages, including genes involved in central metabolism (*pykF*, *ppc, gltA*, *spoT*, and *slr2124*); transcription (*rpoC1*, *sigA*, *sigC*, and *kaiC*-*like protein* 1); translation (*fusB*)*;* photosynthesis and respiration (*ndhF*); chemotaxis (*mcpA*); and genes of unknown function (*slr0753*, *slr1546*, *slr6022*/*slr6081*, and *ssr5117*). As proof of concept, the authors introduced representative mutations in the genes *ndhF1* (NdhF1-F124L strain) and *fusB* (EF-G2-R461C strain). Both of the single mutations conferred enhanced growth, final dry mass, and higher carotenoid content under 700 μmol photons m^−2^ s^−1^ compared with the non-tolerant laboratory starting strain, highlighting that distinct pathways can mediate high light adaptation. Each mutant exhibited a distinct adaptive mechanism, with NdhF1-F124L increasing cyclic electron flow and respiration. In contrast, EF-G2-R461C reduced cyclic electron flow but achieved tolerance by preserving the steady-state levels of photosystem protein complexes, particularly PSII [48].

Yoshikawa et al. [49] provided further insights into the transcriptomic shifts underlying evolutionary adaptation. The authors demonstrated that the high light tolerance of the evolved strain remained stable, even after 15 days of serial subculturing under low-intensity light conditions. According to the authors, this persistence suggests that the phenotype was associated with stable genomic changes rather than being solely attributed to reversible physiological acclimation. Comparative transcriptomic analysis revealed a clear divergence between the parental and evolved strains. Whereas the parental strain showed minimal expression changes between moderate- and high-intensity light (4000 and 7000 μmol photons m^−2^ s^−1^), approximately 28% of genes showed differential expression between the parental and the adapted strain at 7000 μmol m^−2^ s^−1^. A hallmark of this adaptive response was the upregulation of the *isiA* and *isiB* genes. The role of *isiA* in photoprotection and stabilization of the photosystem I (PSI) supercomplex is well established, whereas the specific contribution of *isiB* to high light tolerance remains less clear.

The observation that *isiA* overexpression in the wild-type strain only partially reproduced the adapted strain phenotype indicates that additional mechanisms, including mutations in *hik26* and *slr1916*, contribute to high-intensity light tolerance [49]. Reverse-engineering experiments further support the functional roles of the evolved *hik26* and *slr1916* alleles in this phenotype [49].

Beyond constant high-intensity irradiance, fluctuating light, characterized by the rapid alternation between high and low intensities on the timescale of minutes, as commonly experienced in outdoor or shaded cultivation, poses a distinct photosynthetic challenge. Unlike sustained high light, fluctuating light disrupts carbon assimilation and damages the photosystems through different physiological mechanisms. Figueroa-Gonzalez et al. [45] applied ALE to *Synechocystis* under two progressively intensified fluctuating-light regimes; the lethal regime additionally featured increasingly shorter low-light intervals. One regime remained non-lethal, whereas the other reached conditions that were lethal to the ancestral strain. This approach generated 24 monoclonal evolved isolates harboring 44 fully segregated mutations absent from the ancestral LT and WT, 28 of which affected proteins or structural RNAs. Two mutations, in *pam68* (involved in photosystem II [PSII] assembly) and *sll0518*, were present in all 24 evolved isolates, suggesting that they emerged early during selection. When individually reintroduced into the fluctuating-light-sensitive ancestral background, each mutation increased tolerance to non-lethal fluctuating light. A gain-of-function mutation in *rpaB* (regulator of phycobilisome association B), recovered independently in three isolates tolerant to the lethal regime, significantly increased tolerance to both lethal fluctuating light and constant high light when reintroduced into the ancestral background. This phenotype was associated with coordinated downregulation of light harvesting and changes in photosystem stoichiometry.

#### 5.1.2. Improving Salt Tolerance

The transition from freshwater to saline media or seawater-based cultivation is a critical bottleneck for the industrial scalability of *Synechocystis*, as large-scale production demands sustainable alternatives to freshwater resources [50]. Beyond enabling seawater-based cultivation, hypersaline environments can promote the synthesis of valuable metabolites, including carotenoids, polyunsaturated fatty acids, and neutral lipids, while reducing the risk of contamination [64,65]. Standard laboratory strains of *Synechocystis* are susceptible to osmotic and ionic stress, necessitating metabolic and physiological reconfiguration to enhance halotolerance, a goal that can be effectively achieved through ALE.

Cyanobacterial strategies for osmotic homeostasis typically involve the active efflux of ions and the intracellular accumulation of compatible solutes, primarily glucosyl glycerol, glucosyl glycerate, and sucrose [64]. To enhance intrinsic salt tolerance, Hu et al. [50] conducted a long-term ALE experiment with *Synechocystis* exposed to 3% NaCl for almost one year, yielding strains with improved growth under salt stress. These evolved populations exhibited not only higher specific growth rates and cell densities but also a distinct morphology characterized by reduced cell diameter, likely reflecting more frequent cell division and a higher surface-to-volume ratio, which may enhance ion efflux [50].

Metabolomic profiling identified 58 intracellular metabolites and revealed two distinct metabolic subclusters among the evolved strains under salt stress, suggesting divergent metabolic responses to salinity. This interpretation was further supported by transcriptomic analyses [50]. Representative strains from each metabolic subcluster displayed unique gene expression patterns associated with salt tolerance. The representative strain selected from one subcluster underwent broad transcriptional reprogramming involving 132 significantly differentially expressed genes, upregulating genes involved in photosynthesis, potassium transport, and oxidative stress defense. Among them, the potassium transporter KtrA and the potassium-transporting ATPase *α* subunit were among the significantly upregulated candidate genes, which is consistent with a role in maintaining ionic balance, although this study did not include reverse-genetic validation. Furthermore, this group exhibited significant upregulation of several functional categories, including genes encoding molybdenum cofactor biosynthesis proteins, ribosome-related proteins, photosystem components, high-light-inducible proteins, and peptide-binding proteins.

In contrast, representative strains from the second metabolic subcluster exhibited more conservative transcriptional responses consisting of only 12 significantly differentially expressed genes. These responses mainly involved genes related to nutrient sensing and peptide binding, including downregulation of glutamine synthetase and the RNA polymerase *σ* factor, alongside the significant upregulation of a gene encoding a peptide-binding protein. Therefore, these results indicate that the metabolically distinct subclusters rely on different transcriptional strategies to cope with salt stress, reflecting divergent metabolic and transcriptional responses under the same selective pressure [50].

More recently, Zhu et al. [51] extended halotolerance engineering beyond the moderate salinity used by Hu et al. [50] by evolving *Synechocystis* under stepwise increases in NaCl from an initial tolerance threshold of 4.0% up to 6.5% (*w*/*v*), yielding four evolved clonal isolates. The authors did not specify whether these isolates originated from independent evolutionary lineages or from a single endpoint population. Whole-genome resequencing identified eight mutated loci relative to the parental strain (three SNPs: *sll1867*, *slr1670*, *sll0689*, and five small insertions/deletions: *slr1753*, *sll1755*, *sll2011*, *sll0377*, and *sll0762*). Systematic knockout, complementation, and overexpression analyses identified *slr1670*, which was hypothesized to encode a glucosyl glycerol (GG) lyase, and *slr1753* as the principal contributors to the evolved phenotype. Disruption of *slr1670* increased intracellular GG accumulation by over 260% and enhanced salt tolerance, which is consistent with the established role of GG as a key compatible solute in *Synechocystis* osmoadaptation. Conversely, overexpression of the evolved *slr1753* allele increased Na^+^ adsorption on the cell surface and thereby reduced residual Na^+^ by 6.4% in simulated seawater, suggesting a potential application in seawater desalination. In contrast, disruption of *sll0689* (encoding a Na^+^/H^+^ antiporter) impaired growth even under salt-free conditions, highlighting the essential role of Na^+^/H^+^ homeostasis for both normal growth and salt adaptation. Beyond mechanistic insights, inoculation with the evolved strain improved the germination and growth of *Brassica rapa chinensis* in saline–alkali soil relative to both untreated and wild-type-inoculated controls, demonstrating the potential of ALE-derived halotolerant *Synechocystis* strains for saline–alkali soil bioremediation [51].

#### 5.1.3. Non-Essential Metal Tolerance

The environmental release of non-essential metal cations poses a severe environmental and health risk due to their persistence, bioaccumulation, and toxicity [6,66]. Among these, Cd^2+^ stands out as a particularly hazardous pollutant, primarily released from industrial activities such as nickel–cadmium battery manufacturing and pigment production, leading to severe water pollution [66]. In industrial hotspots, such as electronic waste recycling facilities, Cd^2+^ concentration in surface water can reach 14.8 µM [67], thus significantly exceeding the threshold reported to inhibit the growth of *Synechocystis* [68].

As one of the most ecologically important groups of prokaryotes, cyanobacteria play a pivotal role in the global carbon cycle [69]. More recently, their potential has expanded toward sustainable bioprocessing in contaminated media, acting as biological agents for the adsorption and sequestration of toxic ions in wastewater purification. However, their practical application is limited by the cytotoxicity of the effluents themselves. Exposure to excessive Cd^2+^ severely affects cellular physiology in cyanobacteria by disturbing photosynthetic activity, nutrient uptake, and redox balance [70,71,72].

To overcome the inherent metal sensitivity, ALE provides a powerful strategy to enhance Cd^2+^ tolerance. By serially passaging a wild-type strain in medium with incremental Cd^2+^ concentrations (Table 1), Xu et al. [52] isolated an evolved strain that exhibited markedly enhanced growth under 9.0 µM CdSO_4_, which is a concentration that typically inhibits wild-type growth. Under standard growth conditions without Cd^2+^, the evolved lineage showed only a slight reduction in growth and a yellow–green coloration, indicating minimal fitness cost. Interestingly, under control conditions, the phycocyanin content was reduced. In contrast, carotenoid content increased in the evolved strain, suggesting that the adaptive response to Cd^2+^ involved modifications in photosynthetic pigment composition. In contrast, Arunakumara and Zhang [6] observed a decrease in both pigments after short-term Cd^2+^ exposure, highlighting that pigment responses may differ substantially between acute stress and long-term adaptation. These findings suggest that prolonged selective pressure during ALE induces metabolic reprogramming that improves oxidative stress management, which is consistent with the known protective role of carotenoids against ROS [68,73].

Whole-genome resequencing of evolved isolates identified nine mutations relative to the wild-type genome (seven non-synonymous SNPs, one structural variation, and one insertion in a low-complexity region). Functional analyses supported the involvement of six loci in Cd^2+^ tolerance (*slr0454*, *slr0623*, *slr0721*, *slr0774*, *slr0798*, and *slr1753*), as disruption of these genes increased Cd^2+^ sensitivity in either the wild-type or evolved background. Among these, the 659-bp deletion truncating *slr0454* showed the strongest evidence for a direct adaptive effect, as overexpression of the evolved allele conferred greater Cd^2+^ tolerance than the wild-type allele. In addition, *slr0798* exhibited the strongest transcriptional induction under Cd^2+^ exposure (up to 36.5-fold). The remaining three mutations (*slr1302*, *ssr1480*, and *sll1586*) showed inconsistent or no functional support. Disruption of *slr1302* impaired growth independently of Cd^2+^ stress, confounding interpretation. Knockout of *ssr1480* increased Cd^2+^ sensitivity in the wild-type background but had no effect in the evolved background, and overexpression reduced tolerance. *sll1586* showed no detectable knockout phenotype in either background despite transcriptional induction [52].

The distinct sets of genes required for resistance in parental and evolved strains further suggest that adaptive evolution under Cd^2+^ stress reshapes the underlying regulatory networks, rather than enhancing a single conserved tolerance pathway. In addition, adaptive evolution under Cd^2+^ stress also conferred cross-tolerance to ZnSO_4_ and CoCl_2_, and better acclimation to higher illumination intensity at 200 μmol photons m^−2^ s^−1^ than the wild-type strain [52]. Although these findings provide valuable insights into the genetic basis of Cd^2+^ tolerance, the study did not directly assess the capacity of *Synechocystis* strains to remove or sequester it from the growth medium. Although the evolved strain exhibits enhanced survival and growth under elevated Cd^2+^ concentrations, the extent to which this strain contributes to actual bioremediation remains unresolved and warrants further investigation.

Building on this evolved strain framework, Xiong et al. [53] investigated whether tolerance to Cd^2+^ and high light could be jointly enhanced by ALE. Starting from a *Synechocystis* strain previously adapted to tolerate over 600 μmol photons m^−2^ s^−1^ of light, the authors performed a second round of ALE under constant high-light conditions while stepwise increasing the Cd^2+^ concentration. This strategy increased Cd^2+^ tolerance from 3.0 to 5.5 mM over approximately 800 days, representing an 83% improvement relative to the high-light-adapted parental strain. Whole-genome resequencing of three independently evolved isolates identified 15 mutated loci. Subsequent gene knockout and complementation analyses demonstrated that four loci (*ssl2615*, *sll1732*, *ssr1480*, and *sll1659*) contributed to Cd^2+^ tolerance under high-light conditions (the first three positively, while *sll1659* acted as a negative regulator). *ssl2615*, encoding the ATP synthase F_0_ subunit c, and *sll1732*, encoding a NAD(P)H-quinone oxidoreductase subunit involved in cyclic electron flow, showed the strongest causal evidence. Importantly, these adaptive loci only partially overlapped with those identified by Xu et al. [52] during ALE under Cd^2+^ stress alone, suggesting that sequential adaptation to multiple stressors is not simply additive but involves a partially distinct genetic basis.

#### 5.1.4. Evolving Acid Resistance

Another industrially relevant trait is tolerance to acidic environments. The acidification of lakes and streams is primarily caused by the anthropogenic emission of sulfur dioxide (SO_2_) and nitrogen oxides (NO_x_). Acidification is emerging as a persistent environmental threat, particularly in the context of increasing energy demands driven by rapid industrialization and urban expansion [74]. These pollutants react with atmospheric moisture to form acid rain (pH < 5.6), which negatively affects ecosystems, soil chemistry, microbial communities, plant physiology, carbon cycling, and human health [54,74,75].

Although mitigation strategies have reduced acid rain severity in some regions, its persistence continues to limit the industrial use of affected environments. ALE provides a strategy to overcome this limitation by allowing microorganisms, such as cyanobacteria, to adapt to low pH conditions. Through ALE, strains can acquire genetic changes associated with improved growth and tolerance under acidic conditions, although whether these adaptations improve productivity or process performance requires separate validation. To elucidate the genetic basis of acid resilience, Uchiyama et al. [54] utilized ALE to evolve strains capable of growth at pH 5.5, a condition that typically inhibits the growth of the parental strain. Whole-genome resequencing of two independent parallel lineages identified eight and nine mutations, respectively, relative to the parental strain (11 distinct mutated genes/positions in total). Both lineages shared two mutations (*sll1951* and *sll0914*), whereas the FoF_1_-ATPase operon was independently targeted through mutations affecting different subunits (*sll1321* and *sll1322*), representing a clear example of convergent evolution at the same functional complex. Quantitative RT-PCR further showed differential expression of a subset of the corresponding genes, including *sll0914*, under acid stress. Because this study did not include knockout, complementation, or allele-reconstruction experiments, the contribution of individual mutations to acid tolerance therefore remains at the level of candidate association, with replicated convergence at the FoF_1_-ATPase operon providing evidence for adaptive relevance. Accordingly, no individual locus is interpreted here as having a definitive causal role in acid tolerance [54].

#### 5.1.5. High Temperature Tolerance

Elevated cultivation temperature represents an additional stressor relevant to outdoor cultivation of *Synechocystis* in tropical and subtropical regions, where daytime culture temperatures can substantially exceed the ~30 °C typically used in the laboratory and approach the 40–45 °C range, above which growth rapidly declines. Tillich et al. [55] applied a mutagenesis-assisted ALE protocol to generate a polyclonal thermotolerant strain mixture (mutIV-mix) capable of stable growth at 45 °C. Whole-genome resequencing of monoclonal isolates derived from this mixture identified recurrent mutations in eight candidate genes, including *clpC* (a chaperone/protease subunit independently mutated at multiple distinct positions across isolates), *pnp* (polynucleotide phosphorylase), *pyk2* (pyruvate kinase), *sigF* (an RNA polymerase sigma factor), *nlpD*, *pyrR*, *pilJ*, and *cya1* (adenylate cyclase). The recurrence of independent mutations at the same loci across evolved lineages provides convergence-based evidence for their functional relevance. However, because *pyk2* encodes an essential glycolytic enzyme and no reverse-genetic validation (e.g., knockout, complementation, or allelic replacement) was performed for any of the candidate loci, this study establishes candidate associations rather than causally validated determinants of thermotolerance.

Building on this foundation, Wang et al. [56] generated strains tolerant to the combination of high-light and high-temperature stress, which is a condition more representative of outdoor, low-latitude cultivation than either stressor alone. The resulting evolved strains exhibited lower reactive oxygen species accumulation, improved photosynthetic performance and higher pigment retention, and greater dry biomass and glycogen content than the wild-type strain under combined high-temperature/high-light conditions. Whole-genome resequencing identified only six mutations across the three evolved strains. Systematic knockout, CRISPR interference, overexpression, and in situ gene-replacement analyses in the parental background identified *slr1329*, encoding the AtpB subunit of ATP synthase, as the locus most clearly contributing to thermotolerance; *sll1626*, encoding the SOS response regulator LexA (despite being highlighted as causal in the original study’s own summary), did not show a consistent growth advantage upon gene replacement, and *sll1457* showed only a weaker, partial positive effect. Comparative transcriptomics further suggested that the thermotolerant phenotype of at least one evolved strain involved coordinated upregulation of oxidative phosphorylation genes and downregulation of peptidase and protease-inhibitor pathways. The mutational spectra of the two studies show no obvious gene-level overlap, suggesting that thermotolerance in *Synechocystis*, similar to tolerance to the other environmental stressors reviewed here, is polygenic and can be achieved through multiple distinct genetic routes.

#### 5.1.6. Tolerance to Environmental Pollutants

Most ALE studies reviewed here apply a single stressor with progressively increasing intensity. Hou et al. [57] instead examined how chronic exposure to environmentally relevant concentrations of either copper (Cu^2+^) or the fluoroquinolone antibiotic moxifloxacin shapes tolerance evolution in *Synechocystis*, modeling long-term pollutant exposure in aquatic environments rather than conventional stepwise stress escalation. Whole-genome resequencing identified distinct stressor-specific *rpoB* mutations, encoding the β subunit of RNA polymerase, together with large tandem duplications and plasmid copy number gains. Population-level resequencing and transcriptomic analyses associated the *rpoB* mutations with tolerance to the selecting stressor. In contrast, the structural variants were associated with a distinct, broad-spectrum cross-tolerance phenotype affecting several non-selecting antibiotics; unlike the rpoB-linked tolerance, this cross-tolerance proved transient, reverting to baseline sensitivity after a 30-day recovery period without stressor exposure.

Wu et al. [58] combined a conventional ALE approach with a separate genome-wide hypermutation strategy to investigate resistance to the antimicrobial pollutant triclosan in *Synechocystis*. The evolved strain showed a 1.40-fold improvement in triclosan tolerance over the wild-type strain. Whole-genome resequencing identified a missense mutation in *fabI* (encoding enoyl-ACP reductase). Molecular docking predicted reduced binding affinity of FabI for triclosan, while reconstruction of this mutation in the wild-type background demonstrated enhanced triclosan tolerance and was associated with a shift in membrane fatty acid composition toward a higher saturated-to-unsaturated ratio.

#### 5.1.7. Adaptation to Wastewater Cultivation

Beyond tolerance to defined chemical or physical stressors, ALE has also been used to adapt *Synechocystis* to a fundamentally different cultivation medium: nutrient-rich wastewater, which offers the dual benefit of reducing freshwater consumption and removing nitrogen and phosphorus from effluent. Hu et al. [59] applied ALE to *Synechocystis* in synthetic wastewater over 374 days of cumulative selection, yielding an evolved strain that reached higher final optical density and dry weight than the wild-type strain by day 13 of cultivation, while removing 35.55% of total nitrogen and 60.95% of total phosphorus from the medium. RNA sequencing identified 165 differentially expressed genes between the evolved and wild-type strains, with photosynthesis-related pathways—including light-harvesting complexes, phycobilisomes, and photosystem components—among the most strongly enriched, which is consistent with the measured increase in photosynthetic oxygen evolution in the evolved strain. The evolved strain also showed a shift in fatty acid composition toward a higher proportion of polyunsaturated fatty acids, which affected biodiesel-relevant indices, including oxidation stability and saponification number. However, whole-genome resequencing was not performed in this study; therefore, the genetic basis underlying the improved growth phenotype remains undetermined.

### 5.2. Tolerance to Products

Another key requirement for developing production strains is the ability of *Synechocystis* to tolerate the accumulation of target compounds during biosynthesis. Products generated endogenously or through heterologous pathways can negatively affect cellular physiology, limiting productivity at higher titers. ALE, particularly when combined with targeted genetic engineering strategies, can be used to obtain strains with improved tolerance to these compounds, supporting more robust and efficient product formation.

#### 5.2.1. Tolerance to Toxic Amino Acids

Aromatic amino acids (AAAs), such as phenylalanine (Phe), tyrosine (Tyr), and tryptophan (Trp), are increasingly in demand for animal feed and as precursors in the pharmaceutical, food, and cosmetic industries [76,77,78]. In *Synechocystis*, these compounds are synthesized via the shikimate pathway, which is a key metabolic route that links carbon metabolism to the production of essential protein building blocks and specialized phenylpropanoids [60]. Unlike heterotrophic platforms, cyanobacteria offer the advantage of converting CO_2_ directly into these precursors using solar energy [76,79].

To overcome the physiological constraints that limit engineered metabolic overproduction, ALE was employed to enhance tolerance. For example, Kukil et al. [60] demonstrated that by subjecting *Synechocystis* to stepwise increases in extracellular Phe, lineages could be evolved to tolerate up to 12 mM of the amino acid. These adapted strains not only sustained robust growth but also shifted from Phe consumers to Phe producers. Under small-scale high-density cultivation (HDC), PRM8 reached a Phe titer of 610 ± 196 mg L^−1^ after 4 days, with a specific productivity of 24.9 ± 7 mg L^−1^ OD_750_^−1^, which is at least sixfold higher than that of the other tested PRMs.

Selection for growth under increasing Phe concentrations was accompanied by mutations in the *ccmA* gene, encoding 3-deoxy-d-arabinoheptulosonate-7-phosphate synthase (DAHPS), specifically within the N-terminal ferredoxin-like (FL) regulatory domain. These genetic modifications were proposed to weaken Phe binding, thereby relieving allosteric inhibition of DAHPS, although this deregulation was associated with reduced catalytic activity in most mutants. The evolved phenylalanine-tolerant lineage also enhanced downstream phenylpropanoid production: expressing phenylalanine ammonia lyase in this evolved background increased trans-cinnamic acid productivity by more than 1.5-fold relative to the non-evolved control, while expressing tyrosine ammonia lyase in the same background increased *p*-coumaric acid productivity more than threefold [60].

Beyond metabolic engineering, ALE has been used to explore the evolutionary resilience of cyanobacteria in biochemically rich environments, mimicking early endosymbiotic stages. Hosoda et al. [61] evolved *Synechocystis* to tolerate a cocktail of six toxic amino acids (Arg, His, Lys, Met, Phe, and Thr). Interestingly, mutations in *ccmA* again emerged, highlighting this locus for adaptation. While Kukil et al. [60] associated *ccmA* mutations with deregulation of the shikimate pathway, Hosoda et al. [61] suggested a potential role in optimizing CO_2_-concentrating mechanisms and photosynthetic efficiency. This divergence underscores how the same genetic target can be recruited to solve different environmental impositions, ranging from metabolic toxicity to the coordination of autotrophic growth in complex nutrient landscapes.

The identification of additional mutations in membrane-related genes, such as those encoding the BgtB subunit of the ABC-type permease for basic amino acids and glutamine, and penicillin-binding protein, further indicates that adaptation to toxic amino acids is a multilayered process [61]. This process not only involves the “rewiring” of internal enzymatic control but also the reinforcement of the cell envelope to modulate metabolite penetration [61].

#### 5.2.2. Biofuel Tolerance

The transition from fossil to renewable fuels requires developing microbial platforms capable of synthesizing sustainable fuels from non-petrochemical resources [80,81]. Among these, the direct conversion of CO_2_ and solar energy into liquid biofuels by photosynthetic microorganisms is often presented as advantageous over traditional lignocellulosic routes, as it requires only simple nutrient inputs and can thrive in areas that cannot support agriculture, while avoiding competition with agricultural land used for food production [2]. *Synechocystis* has been genetically modified to produce various biofuels, including isobutanol, butanol, ethanol, alkanes, alkenes, biodiesel, and hydrogen [82,83,84,85]. Industrial-scale implementation remains hampered by the acute cytotoxicity of these end-products [28]. Consequently, the economic feasibility of large-scale biofuel production by cyanobacteria requires not only metabolic flux optimization, which is typically achieved through the heterologous expression of enzymes from native producers, but also the development of strains with enhanced robustness to solvent-induced stress via ALE [86,87].

Among the various candidates for supplementing gasoline, n-butanol and isobutanol are particularly promising due to their low hygroscopicity, low flammability, and energy content (27 MJ/L), which is similar to gasoline (32 MJ/L). Despite their similarities, these alcohols differ in their carbon backbone structure, with n-butanol being linear and isobutanol branched, which is a distinction that influences both their industrial application and toxicity profile [88]. Notably, n-butanol and isobutanol impose a significant physiological burden by damaging cell structure and function, thereby inhibiting growth [28]. To elucidate the systemic changes necessary to enhance robustness, Wang et al. [1] conducted a 395-day ALE experiment that increased n-butanol tolerance from 0.2% to 0.5% (*v*/*v*), corresponding to a 150% increase in the tolerated concentration. Metabolomic analysis of the evolved strains revealed that adaptation to this linear isomer was driven by shifts in nine key metabolites, including *D*-fructose-6-phosphate (F6P), NADPH, *D*-(-)-3-phosphoglyceric acid (3PG), phosphoenolpyruvic acid (PEP), *D*-glucose-6-phosphate (G6P), *D*-ribose-5-phosphate (R5P), glycerol, stearic acid, and serine. These findings suggest a shift in the central carbon metabolism and lipid biosynthesis to stabilize the cell membrane under n-butanol stress. Consistent with roles reported in other stress contexts, glycerol and stearic acid may contribute to membrane remodeling, while serine and other amino acids may support broader stress resistance [1].

Similarly, to enhance isobutanol tolerance in the glucose-tolerant *Synechocystis* strain, Matsusako and coauthors [40] employed ALE to isolate evolved strains capable of growing at 5 g L^−1^ of isobutanol, which is a concentration that completely inhibited the wild-type strain. Notably, the evolved strains no longer exhibited the cell aggregation typically observed in the parental culture under isobutanol stress, indicating additional phenotypic adaptations associated with enhanced tolerance [40]. Genomic analysis identified a recurring mutational landscape, characterized by the consistent mutation of *envD* (a resistance-nodulation-cell division family efflux protein) and a disruption of genes associated with pili formation, such as *mcpA* (a methyl-accepting chemotaxis protein) and *hik43* (a two-component hybrid sensor and regulator). Reverse engineering confirmed that the simultaneous inactivation of *mcpA* and *envD*, or *hik43* and *envD*, conferred isobutanol tolerance through a synergistic effect, as the double-deletion mutants displayed growth patterns similar to those of the evolved strains [40]. Additionally, the tolerant strains exhibited cross-resistance not only to isobutanol but also to a wide variety of alcohols such as ethanol, n-butanol, and isopentanol, which is likely due to the combinatorial mutations in *mcpA* and *envD*, or *hik43* and *envD* [40].

Enhanced tolerance traits obtained in such evolved strains provide a starting point for combination with targeted genetic engineering. However, this strategy has so far been quantitatively validated at the productivity level only in the isobutanol/ethanol system described above. Broader claims of industrial-scale benefit will require further studies that couple ALE-derived tolerance with engineered production pathways and report titer, yield, and productivity under process-relevant conditions.

Critically, Matsusako et al. [40] went beyond a tolerance phenotype to demonstrate a direct productivity benefit. The introduction of a heterologous ethanol-producing pathway (*pdc/adhII* from *Zymomonas mobilis*) into the alcohol-tolerant evolved background increased ethanol productivity to 142% of that of the same pathway in the non-evolved parental strain (14.8 ± 0.2 vs. 10.4 ± 0.4 mg L^−1^ OD^−1^ at 72 h).

### 5.3. ALE Combined with Metabolic Engineering

Beyond selecting for tolerance in an otherwise unmodified genetic background, ALE can also be applied downstream of, or interleaved with, targeted metabolic engineering, either to rescue a genotype rendered non-viable by an engineered deletion or to improve the performance of an already-engineered production strain. Two recent studies illustrate this hybrid design in *Synechocystis*.

Ludwiczak et al. [62] addressed a fundamental question in photosynthetic electron transport: whether oxygenic photoautotrophic growth can occur in the complete absence of photosystem I (PSI). Starting from a PSI-deficient background in which all twelve native PSI genes had been replaced by their *Arabidopsis* counterparts, with no detectable PSI subunits from either the endogenous or the introduced plant genes, the authors obtained evolved lineages capable of sustained photoautotrophic growth despite lacking detectable PSI subunits from either *Synechocystis* or the plant transgene. Whole-genome resequencing of the eight evolved lineages identified recurrent, independently arising nonsynonymous mutations in *fusA* (*slr1463*, encoding elongation factor G) in seven of the eight lineages, comprising nine distinct alleles. The remaining lineage carried a mutation in *lmbP* (*slr0862*, a gene of the GHMP kinase superfamily), which also segregated in three additional evolved populations, suggesting that both genes represent recurrent adaptive targets.

Reintroducing two of the recurrent *fusA* alleles (*fusA*^I28T^ and *fusA*^P307L^) individually into their respective non-evolved, PSI-deficient parental strains restored photoautotrophic growth, providing direct causal validation of their contribution to the evolved phenotype. However, introducing *fusA*^I28T^ into a newly constructed Δ*psaAB* strain lacking the additional background mutations accumulated during evolution failed to restore autotrophy. Only after a second round of ALE involving more than 100 independent replicate cultures propagated for an additional ~15–16 weeks did three lineages regain the phenotype. Together, these experiments demonstrate that *fusA* mutations are necessary but not sufficient to restore PSI-independent photoautotrophy in a naïve Δ*psaAB* background, revealing an epistatic requirement for at least one additional adaptive mutation.

Further analyses strengthened this conclusion. Disruption of *ndhB*, which is required for the assembly of the NDH-1 complex, abolished the evolved photoautotrophic phenotype. Notably, complete segregation of the knockout could not be achieved in the evolved background, indicating strong selection against loss of *ndhB*. In contrast, deletion of *pgr5*, which supports an alternative cyclic electron transport pathway, was dispensable for photoautotrophic growth, although it reduced the growth rate. These results implicate reverse electron transport through NDH-1, transferring electrons from plastoquinone to ferredoxin, as the most likely mechanism enabling PSI-independent photoautotrophic growth [62].

Wu et al. [41] applied ALE in a complementary direction. Rather than rescuing a non-viable engineered strain, the authors used salt stress to further improve the performance of an already functional mannitol-producing *Synechocystis* strain. Starting from a compatible-solute-deficient strain engineered to secrete mannitol, ALE generated evolved isolates with enhanced salt tolerance, raising the maximal tolerated NaCl concentration from 300 to 450 mM. The evolved isolates also showed a substantial increase in mannitol production, reaching a specific productivity of 27.7 mg L^−1^ OD_730_^−1^ at 350 mM NaCl, which is approximately 24-fold higher than the parental producer under the same conditions. After 14 days, total mannitol accumulation reached approximately 700 mg L^−1^, compared with ~45 mg L^−1^ in the parental strain.

Whole-genome resequencing identified mutations in only two genes among the prioritized candidates: *pnp* (encoding polynucleotide phosphorylase), which acquired three distinct alleles across the three evolved isolates, and *sigA/rpoD1* (encoding the primary RNA polymerase sigma factor), mutated in one isolate. Reverse engineering provided strong evidence implicating *pnp* in the evolved production phenotype. Partial disruption of *pnp* alone in the parental strain (a complete knockout could not be obtained, which is consistent with the gene being essential) increased mannitol production 6.4-fold, implicating *pnp* as an important contributor to mannitol accumulation. However, this manipulation accounted for only part of the approximately 24-fold increase observed in the evolved isolates. No complementation analysis was performed, and the partial-disruption strain exhibited approximately 30% slower growth and lower salt tolerance than its parental (non-evolved) producer strain [41].

## 6. Knowledge Gaps and Current Challenges

**Figure 3:**
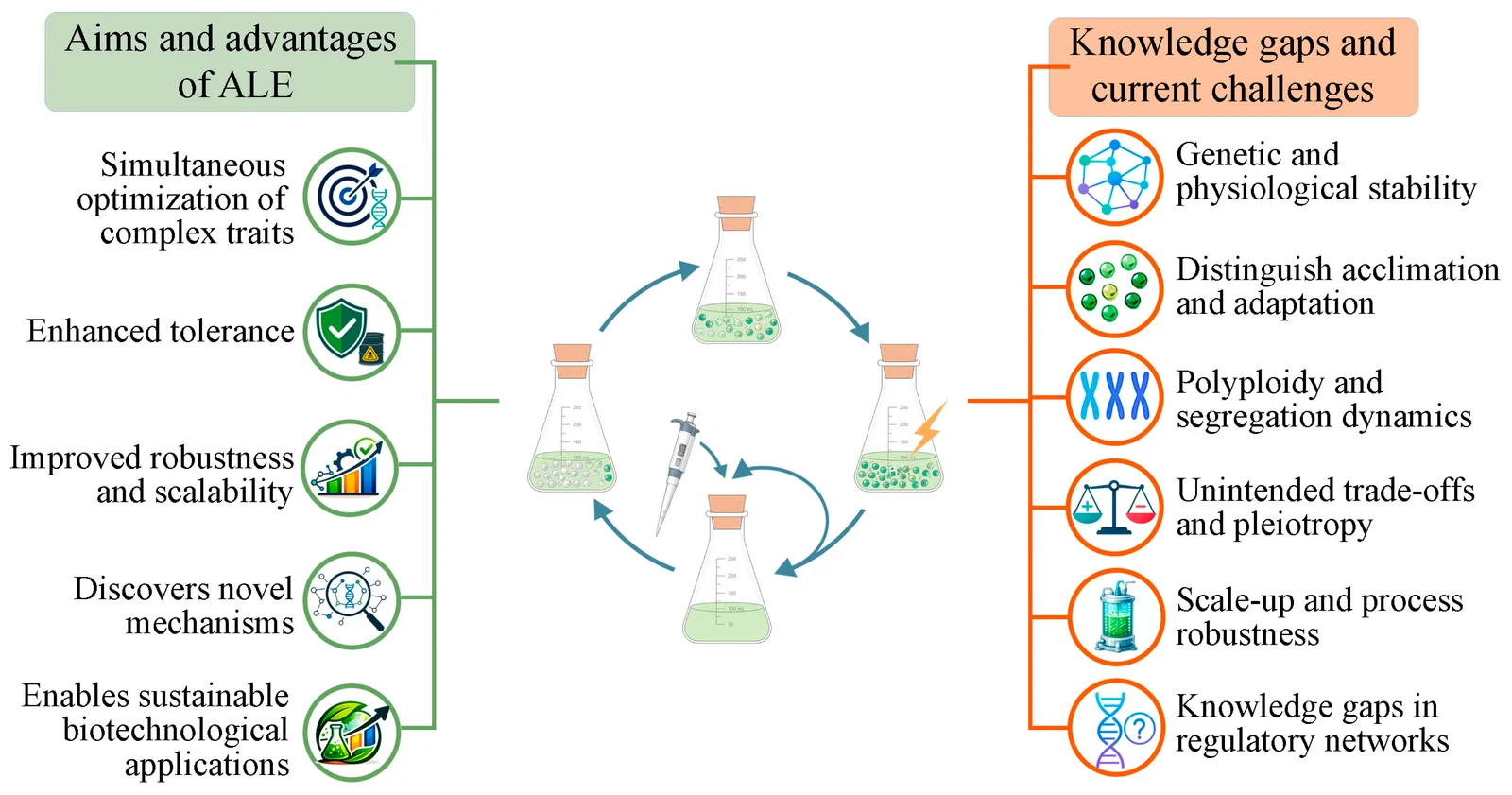
Schematic representation of the aims, advantages, and future challenges of the ALE approach. The central diagram illustrates the iterative ALE cycle of selection and passaging. (**Left**) Key goals and benefits of the ALE approach. (**Right**) Despite the potential of the ALE approach, several knowledge gaps and technical challenges remain.

ALE has emerged as a highly effective strategy for optimizing *Synechocystis* strains, enabling the simultaneous tuning of complex metabolic pathways, enhancing substrate utilization efficiency, and increasing tolerance to toxic compounds or environmental stresses [19]. By selecting for traits that improve cellular robustness and other growth-coupled phenotypes, ALE provides a powerful framework for generating strains with enhanced performance under non-optimal conditions, which is particularly valuable from a large-scale cultivation or biotechnological point of view. Despite these advantages, several bottlenecks continue to hinder the transition from laboratory success to industrial-scale implementation (Figure 3).

One of the most critical and frequently underappreciated challenges in applying ALE-derived strains to industrial processes is ensuring the long-term genetic and physiological stability of the evolved traits. *Synechocystis* presents a particular challenge in this regard, as it exhibits exceptionally high metabolic plasticity and can extensively remodel its transcriptional landscape in response to sustained environmental stress [89]. A strain cultivated under continuous selective pressure may display a markedly improved phenotype. This improved phenotype is not due to beneficial mutations being fixed in the genome, but rather because the strain maintains a stress-adapted transcriptional and metabolic state that is conditional on the continued presence of the selective condition. In such cases, the apparent fitness gain is a phenotypic state, not a heritable genotypic change. Distinguishing these two contributions requires stability assays in which evolved strains are propagated for several generations in the absence of selective pressure. The persistence of the phenotype under these conditions provides strong evidence for genetic adaptation, whereas phenotypic reversion indicates that acclimation was the primary driver [14,90].

Even when adaptation is linked to a genomic basis, attributing the evolved phenotype to specific causal mutations remains a major analytical challenge. ALE often results in the accumulation of multiple interacting mutations and broad regulatory adjustments, which collectively contribute to improved robustness, metabolic efficiency, and stress tolerance. While several studies have successfully employed reverse engineering to identify and validate individual mutations, demonstrating, for example, that a specific single-nucleotide polymorphism in *envD* [40] can enhance tolerance to isobutanol, these targeted validations often fail to capture the full complexity of the evolved phenotype. A complementary and statistically powerful approach for prioritizing causal mutations involves the parallel evolution of multiple independent replicate populations from the same ancestral strain. In this framework, genetic loci that independently accumulate mutations across multiple replicates reflect strong positive selection and are likely to represent adaptive mutations rather than neutral hitchhikers [14,32,91]. This convergence-based approach provides statistical confidence in mutation prioritization without requiring the reconstruction of every candidate individually. Nevertheless, even after identifying the most probable adaptive mutations through convergence analysis and reverse engineering, a residual fraction of the evolved phenotype typically remains unexplained—contributed by epistatic interactions among multiple loci—by compensatory mutations that compensate for the fitness costs of earlier adaptive changes, and by persistent regulatory adjustments that co-evolved with the genomic changes but are not captured by genome sequencing alone [1,14]. This layered complexity means that the phenotype of an ALE-derived strain is not fully encoded in its genome sequence, presenting a fundamental obstacle to rational reconstruction, strain stabilization, and industrial deployment.

In *Synechocystis*, this challenge of phenotypic stability is further compounded by its highly polyploid nature, with reported chromosome copy numbers varying considerably according to growth phase, substrain, and environmental conditions. An early work suggested a count of approximately 12 chromosome copies per cell [34], and an initial real-time PCR study reported considerably higher values of up to 142–218 copies during early exponential growth [92]. However, a subsequent study by the same group, which cross-validated the measurements using two independent quantification methods (real-time PCR and a spectroscopic approach), substantially revised these estimates downward. Genome copy number ranged from approximately 20–27 at low cell density (OD_750_ of 0.1) to approximately 4 at high cell density (OD_750_ of 2.5), with values as low as 1 copy per cell observed under prolonged phosphate starvation and up to 53 copies under reduced light intensity [46]. Regardless of the precise range, it is well established that copy number decreases substantially as cultures progress from low to high density [46,92]. Because the phenotype of a polyploid cell reflects the combined contribution of all its chromosome copies, a beneficial mutation that initially arises in only a subset of chromosomes (i.e., a heteroplasmic state) contributes only part of the total mutant allele dosage until segregation to homoplasmy occurs. Selection acts on whole-cell phenotypes rather than on individual chromosome copies. If the mutant allele dosage is insufficient to confer a selective advantage, or if the adaptive mutation no longer provides a selective advantage once the selective pressure is removed, cells dominated by wild-type chromosome copies may become more frequent over time, leading to an apparent phenotypic reversion even though the mutant allele may persist at a low copy number [93,94]. Consequently, selective pressure should be maintained until beneficial mutations have segregated toward homoplasmy before their stability is evaluated.

This process is mechanistically distinct from the instability of episomal or chromosomally integrated heterologous constructs. As highlighted by Jones [95], genetic instability in cyanobacteria often involves structural changes in engineered DNA, including truncation of heterologous genes or gene silencing arising under metabolic burden. Accordingly, complete segregation of naturally arising point mutations should be verified using quantitative approaches, such as allele-specific qPCR or deep resequencing of clonal isolates, rather than inferred solely from phenotypic stability. Understanding the trade-offs between robustness, productivity, and long-term genetic stability is essential for developing ALE-derived *Synechocystis* strains suitable for future industrial applications [95].

Beyond the challenge of mutational stability, a critical concern involves the unexpected physiological trade-offs associated with adaptive evolution. Evolved *Synechocystis* strains often display side effects that compromise aspects of their original fitness, highlighting the complexity of balancing stress tolerance with overall cellular performance. For example, acid-tolerant strains have been reported to exhibit significantly reduced growth under neutral pH conditions, demonstrating that adaptations conferring resistance to one stress can impair performance under standard conditions [54]. Similarly, the overproduction of protective metabolites, while beneficial under selective pressure, can create metabolic bottlenecks that divert resources away from desired biosynthetic pathways. Cadmium-tolerant strains, for instance, prioritize the synthesis of carotenoids involved in protective mechanisms over phycocyanin assembly, leading to reduced photosynthetic efficiency when grown under non-stress conditions [52]. These observations highlight the potential for pleiotropic effects and resource allocation conflicts in evolved strains, emphasizing the importance of thoroughly characterizing both the benefits and unintended consequences of adaptive evolution, particularly when translating laboratory improvements to industrial-scale production systems.

Translating these laboratory gains into industrial applications requires moving beyond laboratory-scale tolerance metrics toward process-level evaluation. Published techno-economic and life-cycle assessments of engineered *Synechocystis* and cyanobacterial production systems indicate that net economic and environmental performance depends on factors that ALE studies rarely address, including CO_2_ sourcing and delivery, nutrient production, photobioreactor mixing, gas–liquid mass transfer, illumination strategy, harvesting, downstream processing, and product end use (e.g., combustion versus long-term carbon storage) [96,97,98]. Photosynthetic CO_2_ fixation alone does not guarantee that a bioprocess is carbon-neutral or economically competitive. Establishing whether these laboratory improvements translate into economically and environmentally competitive processes requires quantitative techno-economic analysis, life-cycle assessment, and pilot-scale validation. To the best of our knowledge, none of these evaluations have yet been reported for any ALE-derived *Synechocystis* strain.

Looking forward, the next frontier in cyanobacterial engineering lies in the integration of ALE with targeted technologies. Rather than viewing ALE and rational metabolic engineering as separate strategies, their synergy offers a powerful design–evolve–reverse engineer cycle. ALE can be used as a discovery tool to identify novel genetic targets (such as recurrent adaptive targets in *ccmA* or *envD*), which can subsequently be reconstructed in wild-type strains using CRISPR/Cas9 or other genome-editing tools, thereby avoiding the need to repeat lengthy evolution experiments [17]. Furthermore, coupling ALE with high-throughput omics analyses and machine learning-based predictive models is essential for deciphering the complex, polygenic architecture of tolerance traits. This integrated approach has the potential to bridge the gap between evolutionary resilience and high-performance industrial production, enabling the rational design of strains that combine robustness, metabolic efficiency, and sustained productivity under large-scale cultivation conditions.

## 7. Concluding Remarks and Prospects

Although cyanobacteria offer several advantages, such as fast growth compared with plants, high photosynthetic efficiency, genetic tractability, and tolerance to diverse environmental conditions, several limitations still hinder the application of *Synechocystis* in high-efficiency industrial processes. Key challenges include limited robustness under environmental and product-induced stresses, and relatively low productivity compared with established industrial microorganisms. The studies reviewed here, employing ALE to enhance *Synechocystis* tolerance, provide valuable insights into the mechanisms underlying stress resistance, which are essential for translating laboratory findings into economically viable industrial applications. Despite its benefits, ALE has some limitations: evolved strains may exhibit side effects such as loss of original fitness or overproduction of non-target metabolites, and the stability and reversibility of ALE-derived strains are not well characterized [28]. Assessing the long-term stability and potential trade-offs of ALE-evolved strains represents a crucial direction for future research.

Importantly, the tolerance and robustness gains reviewed here have thus far been demonstrated under defined laboratory conditions. Whether these traits translate into improved environmental, energetic, or economic performance at process scale depends on multiple factors, including CO_2_ supply, cultivation conditions, energy inputs, harvesting, and downstream processing, which fall outside the scope of the ALE studies reviewed. To the best of our knowledge, none of the evolved strains discussed here have yet been evaluated through techno-economic analysis, life-cycle assessment, or pilot-/industrial-scale cultivation.

Overall, the evidence reviewed here indicates that ALE has become a powerful complementary strategy to rational metabolic engineering, particularly for improving complex polygenic traits that remain difficult to engineer directly. Continued integration of ALE with genome editing, systems biology, and predictive computational approaches is expected to accelerate the development of robust, high-performing *Synechocystis* strains capable of supporting sustainable industrial biotechnology.

## Data Availability

No new data were created or analyzed in this study. Data sharing is not applicable to this article.

## Supplementary Materials

The following supporting information can be downloaded at: https://www.mdpi.com/article/10.3390/microorganisms14081836/s1, Figure S1: Search flow diagram of literature search. GS, Google Scholar; ALE, adaptive laboratory evolution; Table S1: Search query variants and search outcomes. Searches were conducted in Google Scholar and PubMed using combinations of the keywords “adaptive laboratory evolution”, “ALE”, “adaptive evolution”, “experimental evolution”, “evolutionary engineering”, “tolerance evolution”, “serial passaging”, “long-term selection”, and “genome resequencing”, together with either “Synechocystis” or “Synechocystis sp. PCC6803”; Table S2: Experimental design and cultivation conditions of the ALE studies included in Table 1; Table S3: Genomic outcomes and functional validation of the ALE studies included in Table 1.
