# Supplementary material for: Adaptive Laboratory Evolution in Synechocystis sp. PCC 6803: Current Status and Perspectives
Source: Microorganisms. 2026 Aug 19;14(8):1836. doi: 10.3390/microorganisms14081836 (PMC13515095; doi:10.3390/microorganisms14081836)

Figure S1. Search flow diagram of literature search. GS, Google Scholar; ALE, adaptive laboratory evolution.

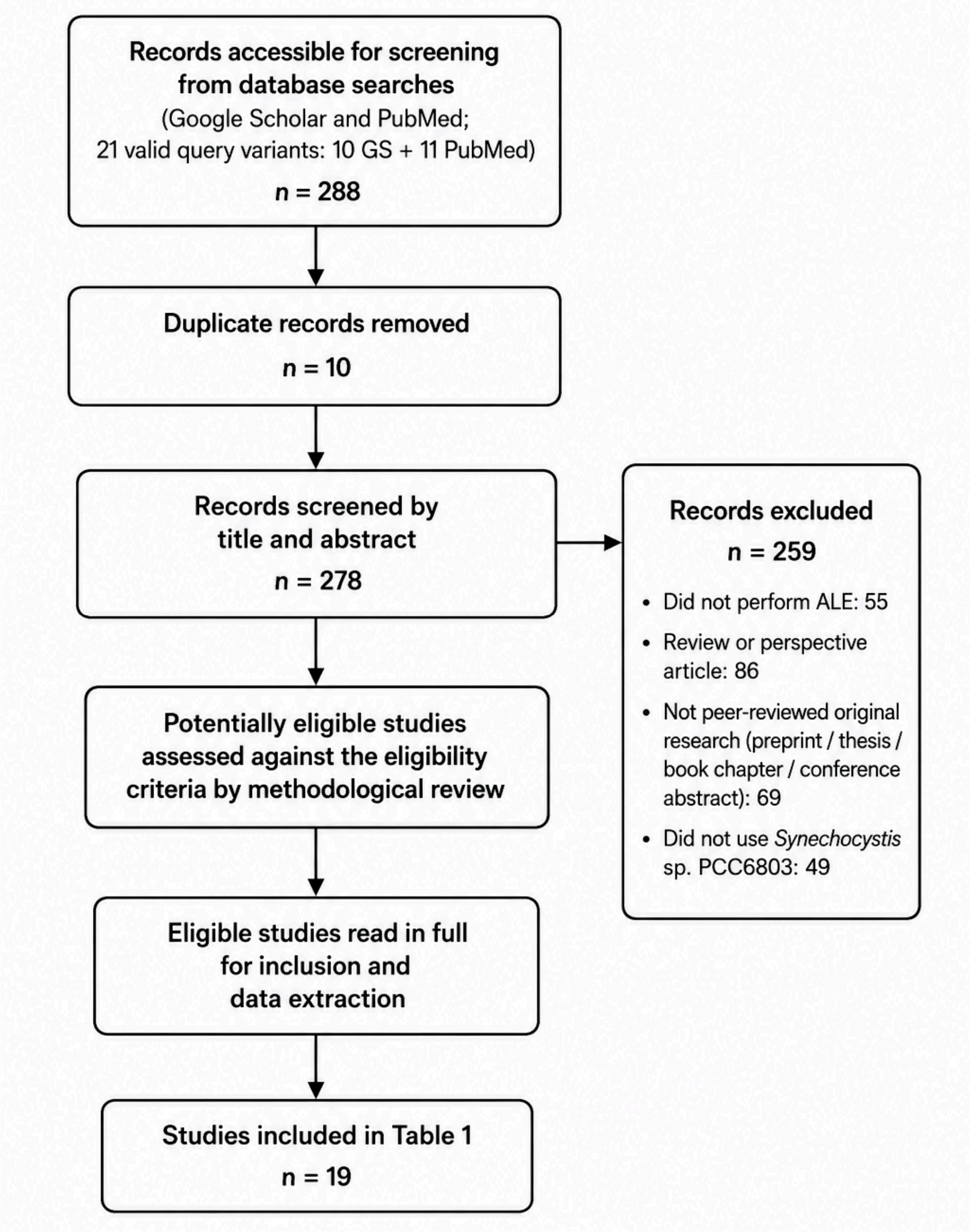

Supplement: Supplementary file 1 [file microorganisms-14-01836-s001.zip › Supplementary Figure S1.pdf]
