# Supplementary material for: Adaptive Laboratory Evolution in Synechocystis sp. PCC 6803: Current Status and Perspectives
Source: Microorganisms. 2026 Aug 19;14(8):1836. doi: 10.3390/microorganisms14081836 (PMC13515095; doi:10.3390/microorganisms14081836)
Supplement: Supplementary file 1 [file microorganisms-14-01836-s001.zip › Supplementary Table S1.pdf]

Table S1. Search query variants and search outcomes. Searches were conducted in Google Scholar and PubMed using combinations of the keywords "adaptive laboratory evolution", "ALE", "adaptive evolution", "experimental evolution", "evolutionary engineering", "tolerance evolution", "serial passaging", "long-term selection", and "genome resequencing", together with either "*Synechocystis*" or "*Synechocystis* sp. PCC6803". No publication-date restriction was applied; only peer-reviewed articles published in English were considered. The table lists all query variants tested, in the order in which they were performed, together with the number of search results displayed by each database and the corresponding search outcomes. One Google Scholar variant (row 5) was discarded as non-reproducible. The remaining 21 query variants comprised 10 Google Scholar and 11 PubMed searches. Searches were last conducted on 10 August 2026. The number of search results displayed by individual queries is not additive because the same records could be retrieved by multiple search variants and because the number of records accessible for screening in Google Scholar may be lower than the estimated number of results displayed. Records accessible for screening were consolidated across the valid searches, yielding 288 records, of which 10 duplicates were removed, leaving 278 unique records for title and abstract screening. Potentially eligible studies were subsequently assessed by reviewing their methodology, and studies meeting the eligibility criteria were read in full for inclusion and data extraction. The final search process identified 19 eligible studies, which are included in **Table 1**.

Query	Database	Search results	Outcome
1. "adaptive laboratory evolution" OR "ALE" OR "adaptive evolution" AND "*Synechocystis* sp. PCC6803"	Google Scholar	≈510	180 results assessed due to partial pagination; 12 potentially relevant studies identified
2. "*Synechocystis*" AND ("adaptive laboratory evolution" OR "ALE" OR "adaptive evolution")	PubMed	14	11 potentially relevant studies identified
3. "adaptive laboratory evolution in *Synechocystis* sp. PCC6803"	Google Scholar	≈180	12 potentially relevant studies identified
4. "ALE" AND "*Synechocystis* sp. PCC6803"	Google Scholar	240	No additional potentially relevant studies identified
5. "adaptive laboratory evolution in *Synechocystis* sp. PCC6803 (no quotation marks)"	Google Scholar	≈14,000	Discarded as non-reproducible (loose term matching rather than phrase matching)
6. "Adaptive Laboratory Evolution" OR "ALE" OR "adaptive evolution" OR "serial passaging" OR "long-term selection" AND "*Synechocystis* sp. PCC6803"	Google Scholar	529	Results screened through page 14 due to practical pagination limits; 19 potentially relevant studies identified
7. "experimental evolution" AND "*Synechocystis*"	Google Scholar	446	Results screened through page 19 due to practical pagination limits; 1 potentially relevant study identified
8. "Experimental evolution" AND "*Synechocystis*"	PubMed	4	3 potentially relevant studies identified
9. "ALE" AND "*Synechocystis*"	PubMed	2	2 potentially relevant studies identified
10. "evolutionary engineering" AND "*Synechocystis*"	PubMed	0	No results
11. "evolutionary engineering" AND "*Synechocystis*"	Google Scholar	326	No additional potentially relevant studies identified
12. "tolerance evolution" AND "*Synechocystis*"	PubMed	0	No results
13. "genome resequencing" AND "*Synechocystis*" AND "evolved"	Google Scholar	35	Results screened through page 9; 1 potentially relevant study identified
14. "genome resequencing" AND "*Synechocystis*" AND "evolved"	PubMed	2	2 potentially relevant studies identified
15. "Adaptive Laboratory Evolution" OR "ALE" OR "adaptive evolution" OR "serial passaging" OR "long-term selection" AND "*Synechocystis* sp. PCC6803"	PubMed	0	No results
16. "Adaptive Laboratory Evolution" AND "*Synechocystis* sp. PCC6803"	Google Scholar	194	19 potentially relevant studies identified
17. "evolutionary engineering" AND "*Synechocystis* sp. PCC6803"	Google Scholar	80	No additional potentially relevant studies identified
18. "tolerance evolution" AND "*Synechocystis*"	Google Scholar	44	2 potentially relevant studies identified

Query	Database	Search results	Outcome
19. "adaptive laboratory evolution" OR "ALE" OR "adaptive evolution" AND "*Synechocystis* sp. PCC6803"	PubMed	0	No results
20. "ALE" AND "*Synechocystis* sp. PCC6803"	PubMed	0	No results
21. "Adaptive Laboratory Evolution" AND "*Synechocystis* sp. PCC6803"	PubMed	0	No results
22. "evolutionary engineering" AND "*Synechocystis* sp. PCC6803"	PubMed	0	No results
