# Supplementary material for: Adaptive Laboratory Evolution in Synechocystis sp. PCC 6803: Current Status and Perspectives
Source: Microorganisms. 2026 Aug 19;14(8):1836. doi: 10.3390/microorganisms14081836 (PMC13515095; doi:10.3390/microorganisms14081836)
Supplement: Supplementary file 1 [file microorganisms-14-01836-s001.zip › Supplementary Table S2.pdf]

Supplementary Table S2. Experimental design and cultivation conditions of the ALE studies included in **Table 1**. Abbreviations: ALE, adaptive laboratory evolution; WT, wild type; GT, glucose tolerant; LT, laboratory-adapted strain; U, one round of UV mutagenesis; M, one round of methyl methanesulfonate (MMS) mutagenesis; lineage names (e.g., UM, UMU, UMMM) indicate the sequential order of UV (U) and MMS (M) mutagenesis rounds; Tol, high light tolerant strain; FL0, non-lethal fluctuating-light regime; FL+, lethal fluctuating-light regime; HL, high light; LL, low light; SNP, single-nucleotide polymorphism; PCA, principal component analysis; WGCNA, weighted gene co-expression network analysis; DEGs, differentially expressed genes; ROS, reactive oxygen species; LED, light-emitting diode.

Characteristics of the evolved strain	Strain	Regime	Lineages	Duration	Vessel	Temperature	Irradiance	CO_2	Transfer / bottleneck	Reference
**Environmental stressors**										
High light tolerance	LT, the laboratory-adapted strain used as the starting strain for ALE, the WT was used as the reference strain	Mutagenesis-assisted up to five mutagenesis rounds (an initial UV treatment followed by up to four additional rounds of UV or MMS) combined with a stepwise increase in light intensity after each round (from 700 to 2300 µmol photons m^{-2} s^{-1})	Nine evolved lineages (U, UM, UMU, UMM, UMUM, UMMM, UMUMM, UMU*, UMU*M) alongside two controls (LT, LT′)	Some lineages maintained for more than 14 months across several rounds of mutagenesis and selection	Multi-Cultivator MC1000-OD tube	23 °C	30 µmol photons m^{-2} s^{-1} for routine conditions; 700 to 2300 µmol photons m^{-2} s^{-1} during ALE	Not specified (ambient aeration)	Cycled from OD_{730} 0.1 to 2.0 (~15 days/cycle, up to 25 cycles) before dilution back to OD_{730} 0.1	Dann et al., 2021 [48]
High light tolerance	GT-I non-motile substrain	Stepwise increase in light intensity from 7000 to 9000 µmol photons m^{-2} s^{-1}; four parallel cultures were used at each transfer	A single evolving population propagated through four parallel cultures at each transfer, with only the fastest-growing culture carried forward at each step	52 days (23 subcultures)	Four parallel test tubes (30 mL modified BG_{11})	34 °C	40 µmol photons m^{-2} s^{-1} for pre-culture/routine conditions; 7000–9000 µmol photons m^{-2} s^{-1} during ALE (point-source LED)	Aerated with sterile air; CO_2 enrichment not specified	Every 2–3 days, the fastest-growing culture among the four parallel tubes was carried forward	Yoshikawa et al., 2021 [49]
Fluctuating-light tolerance	GT *Synechocystis* laboratory type	Progressively intensified alternating high/low light cycles (7–14 days per cycle; HL from 700 to 1200 and LL from 50 to 12 µmol photons m^{-2} s^{-1}), with 3	Six independent populations; 4 monoclonal isolates from each (24 total)	20 selection cycles over 20 months	100 mL glass tubes (70 mL working volume), temperature-controlled multicultivator	23 °C	Alternating high/low light cycles, progressively intensified from HL700/LL50 to HL1200/LL12 µmol photons m^{-2} s^{-1}	Constant aeration; CO_2 enrichment not specified	Cycle length 7–14 days; no explicit OD-based transfer criterion stated	Figueroa-Gonzalez et al., 2026 [45]

Characteristics of the evolved strain	Strain	Regime	Lineages	Duration	Vessel	Temperature	Irradiance	CO_2	Transfer / bottleneck	Reference
		populations under a non-lethal FL0 regime, 3 under a lethal FL+ regime								
Salt tolerance	WT *Synechocystis*; no specific substrain designation given	Constant 3% NaCl selective pressure	Ten independent populations (plus one unstressed control)	Approximately 303 days	12-well culture plates (3 mL culture volume per population)	30 °C	Continuous light, 30 ± 5 µmol photons m^{-2} s^{-1}	Not explicitly stated	Every 7 days, initial OD_{730} set to 0.2	Hu et al., 2020 [50]
Salt tolerance	WT *Synechocystis*; specific source substrain not stated	Stepwise increase from 4.0% to 6.5% NaCl (0.1% increments, with each increase introduced once growth matched the previous step); initial threshold at 4.0% NaCl, corresponding to the wild type's tolerance limit	A single evolved population; four monoclonal isolates (ALE-1 to ALE-4) picked at the endpoint, whether they represent independent sub-lineages or clones of one endpoint population was not stated	Approximately 125 passages over 856 days	Illuminating shaking incubator (160 rpm); exact vessel type not stated	30 °C	50 µmol photons m^{-2} s^{-1}	Not stated	pH 7.5 BG11; step advanced once growth matched the previous step	Zhu et al., 2025 [51]
Cadmium tolerance	WT ATCC 27184	Stepwise increase from 4.6 to 9.0 µM $CdSO_4$	A single evolving lineage; four single colonies picked at the endpoint (fastest-growing designated ALE-9.0); an intermediate isolate ALE-7.0 (sampled at the 7.0 µM stage) also sequenced to trace mutation dynamics	128 passages over 802 days	100 mL shake flask, 20 mL BG_{11}	30 °C	50 µmol photons m^{-2} s^{-1}	Not stated	Concentration raised by 0.3–0.4 µM once growth reached OD750 0.5 (from an initial 0.1) within 96 h	Xu et al., 2018 [52]
Cadmium and high light tolerance	HL-0, a high-light-adapted *Synechocystis* substrain (previously evolved to tolerate >600 µmol photons m^{-2} s^{-1} in an	Stepwise increasing Cd^{2+} ($CdSO_4 \cdot 8/3H_2O$) concentration (3.0–5.5 mM) under constant high light	3 parallel lineages, each in its own shake flask, yielding one evolved isolate per lineage (HLHC-1,	~800 days	100-mL shake flasks, 20 mL BG11 culture volume, 160 r·min^{-1} shaking incubator	30 °C	~600 µmol photons $m^{-2}s^{-1}$ (constant)	Not specified	Cd^{2+} concentration increased by 0.1–0.2 mM whenever the culture grew from OD_{750} = 0.04 to OD_{750} = 0.50 within 5	Xiong et al., 2024 [53]

Characteristics of the evolved strain	Strain	Regime	Lineages	Duration	Vessel	Temperature	Irradiance	CO_2	Transfer / bottleneck	Reference
	unpublished prior study), used as the ALE starting strain		HLHC-2, HLHC-3)						days; upon reaching 5.5 mM, cultures were plated on BG_{11} agar with 5.5 mM Cd^{2+} and the fastest-growing single colony picked from each plate	
Acid tolerance	Laboratory stock *Synechocystis* RK-1 (“parent,” P), carrying 26 mutations relative to the public Kazusa reference genome (background substrain drift)	Stepwise decrease from pH 6.0 to 5.75 and then to 5.5 (1 month per step)	Two independent parallel lineages (A.A.1, A.A.2)	3 months (1 month at each of 3 sequential pH steps)	50 mL BG_{11} (vessel type not further specified)	30 °C	30 µmol photons m^{-2} s^{-1}	3% CO_2-enriched aeration	Weekly transfers, cultures kept below OD_{730} 0.05	Uchiyama et al., 2015 [54]
High temperature tolerance	Parental wild type; genomic differences from the 1996 Kazusa reference were noted	Mutagenesis-assisted (UV + MMS); temperature ramped stepwise together with light	A polyclonal strain mixture (mutIV-mix to mutIV-mix-2); five monoclonal strains (C5K, A1, A6, H2, H12) isolated from 27 (incubator) and 81 (robot) candidates screened, of which only 14 outperformed the wild type	mutIV-mix further selected for one additional year (mutIV-mix-2)	T25 cell culture flasks in a shaking incubator (incubator screening, 27 strains); 96-deepwell plates under robot screening (81 strains); validation of monoclonal strains used a 1 L photobioreactor	Day conditions rose from 28 °C to 47 °C (25 °C night) in the incubator-screening stage; 30 °C to 47 °C (26 °C night) in the deepwell/robot stage; photobioreactor validation performed in increasing temperatures	~19 µmol photons m^{-2} s^{-1} (constant, incubator stage); ~3 rising to ~85 µmol photons m^{-2} s^{-1} (day, deepwell stage); ~125 µmol photons m^{-2} s^{-1} during the day in the photobioreactor stage (dark at night)	0.5% CO_2 (incubator stage); 2% CO_2 (deepwell/robot stage); 10% CO_2/air added periodically, pH 7.3 controlled (photobioreactor stage)	Temperature increased in 0.5 °C increments up to 35 °C, then in maximum increments of 0.2 or 0.1 °C per day depending on culture behavior; each temperature was maintained for 3–7 days for growth-rate determination	Tillich et al., 2014 [55]
High temperature and high light tolerance	HL-4, a high-light-evolved strain from the same group’s prior work, itself derived from wild-type *Synechocystis* (ATCC 27184)	Stepwise temperature increase (from 35 to 37 °C in 1 °C increments, then 37 to 40 °C in 0.5 °C increments) under constant high light	Three independent evolved lineages (HLHT-1/2/3)	81 passages across 700 days	Not stated (transferred to fresh 20 mL cultures)	Stepwise from 35to 40 °C (see Regime); baseline maintenance at 30 °C for both wild type and HL-4 parent	Constant 750 µmol photons m^{-2} s^{-1} during ALE; baseline maintenance at 50 µmol (wild type) or 750 µmol (HL-4 parent)	Not stated	Transferred to fresh 20 mL cultures at OD_{750} 0.04 once growth stabilized at each temperature step	Wang et al., 2026 [56]
Copper and moxifloxacin tolerance	WT *Synechocystis*; specific substrain source not stated	Chronic exposure to a concentration gradient of copper (Cu^{2+}: 10, 50, or 250 µg L^{-1}) or	Three independent replicate populations per treatment (plus an unstressed control);	140 days	250 mL Erlenmeyer flasks (150 mL BG_{11}), 120 rpm orbital shaking	28 ± 1 °C	Continuous illumination, 2000 lx (reported in lux, not µmol photons m^{-2} s^{-1}, as in the original source)	Not stated	~20 weekly serial transfers (3.33% v/v inoculum, initial OD_{730} 0.1) over 140 days	Hou et al., 2025 [57]

Characteristics of the evolved strain	Strain	Regime	Lineages	Duration	Vessel	Temperature	Irradiance	CO_2	Transfer / bottleneck	Reference
		moxifloxacin (0.2, 2, or 20 μg L^{-1}); only the highest concentration of each (250 μg L^{-1} Cu^{2+}, 20 μg L^{-1} moxifloxacin) produced a significant tolerance response and was carried forward	endpoint populations (not single clones) used downstream							
Triclosan tolerance	*Synechocystis*, no specific substrain named	Serial passaging under constant triclosan (0.8 mg L^{-1}; optimized from a preliminary screen of 0.2–1.5 mg L^{-1}), started at 0.6 g L^{-1} initial biomass; cycle 1 extended by 2 days (7 days total) due to an extended lag phase under initial TCS exposure; subsequent cycles held at 5 days each	Triplicate 100 mL cultures; multiple isolates were subsequently sequenced from this lineage (reported as "ALE strains," plural), yielding the named endpoint strain ED30	32 cycles, 162 days total	Not explicitly stated for the formal ALE regimen	Not explicitly stated for the formal ALE regimen	Not explicitly stated for the formal ALE regimen	Not stated	At each 5-day cycle, cultures were centrifuged (6,000 rpm, 10 min), washed twice with sterile deionized H_2O, and resuspended in fresh BG_{11} to 0.6 g L^{-1} biomass; TCS was re-supplemented to 0.8 mg L^{-1} at each transfer	Wu et al., 2026 [58]
Adaptation to wastewater cultivation	*Synechocystis*, maintained as stock on solid BG_{11} (1.5% agar) at 25 °C; no specific substrain named	Repeated cycles of ~10-day cultivation in synthetic wastewater (MBG medium, re-inoculated at OD_{730} = 0.1 each cycle), interspersed with single-colony isolation on BG_{11} agar and liquid expansion in BG_{11} before returning to wastewater	Single lineage; one monocolony randomly selected and expanded after each wastewater cycle, then reintroduced into fresh synthetic wastewater	374 days of cumulative selection in synthetic wastewater, within a total elapsed process of ~2.5 years (the difference reflecting time spent in BG_{11} expansion, monocolony isolation, and contamination-removal stages, which is excluded from the 374-day count)	50 mL synthetic wastewater (MBG) culture in a 200 mL flask	30 °C during the wastewater cultivation stage (stock/plate maintenance was at 25 °C)	Not explicitly stated for the ALE process itself	Not stated	Every ~10 days: culture diluted and spread onto BG_{11} agar to isolate a single colony, which was expanded in liquid BG_{11}, then re-harvested by centrifugation and reintroduced into fresh synthetic wastewater at OD_{730} = 0.1	Hu et al., 2024 [59]
Phenylalanine tolerance	GT *Synechocystis*	Spontaneous-mutant plate selection	Ten independently isolated	Not stated in the primary text	BG11 agar plates, then 24-well and	30 °C	45 μmol photons m^{-2} s^{-1} for routine cultivation;	Supplied via a membrane system (CellDEG	Cultures that yellowed (bleached) were	Kukil et al., 2023 [60]

Characteristics of the evolved strain	Strain	Regime	Lineages	Duration	Vessel	Temperature	Irradiance	CO_2	Transfer / bottleneck	Reference
		(distinct from classic serial-liquid ALE); resistant colonies first selected on BG11 agar with 1 mM phenylalanine, then serially passaged with stepwise increasing phenylalanine, discarding cultures that yellowed and advancing only those that remained green	phenylalanine-resistant colonies (PRM1-12, numbering not continuous); 3 (PRM1, PRM8, PRM12) whole-genome sequenced, remaining 7 assessed by targeted sequencing of all three candidate loci (*ccmA*, *slr1916*, *sll1995)*		later 6-well liquid plates		selection/escalation phase specifically described as 'low light intensity', value not given	HD10), only during the later production-testing stage	discarded; only those that remained green were advanced	
Toxic amino acids tolerance	GT substrain	Stepwise increase in concentration of a six-amino-acid toxic cocktail (TCM_x: Arg, His, Lys, Met, Phe, Thr)	A single evolving population, branching-bottleneck design	85 transfers over 408 days (548 generations at the TCM1 endpoint); TCM_x concentration increased stepwise through day 79, after which transfers continued exclusively in TCM1	50 mL Erlenmeyer flasks, 10 mL cultures	30 °C	10 µmol photons m^{-2} s^{-1} (red:blue LED ratio 9:1)	Not specified	Every 3–4 days (fixed interval); each transfer split into three or four parallel TCM_x concentrations, with the branch reaching the highest x and a population increase >√10-fold (~3.16-fold) carried forward (~2×10^6 cells/mL starting concentration; bottleneck ~2×10^7 cells in 10 mL)	Hosoda et al., 2014 [61]
n-Butanol tolerance	*Synechocystis* WT, no more specific substrain designation confirmed	Stepwise increase from 0.2% to 0.5% v/v butanol (0.05% increments)	Four independent populations	94 passages over 395 days (~700 generations; ~7.5 generations/passage estimated)	250 mL flasks, 50 mL BG_{11}	30 °C	50 µmol photons m^{-2} s^{-1}	Not specified	Transfers at OD_{730} 0.5 (typically every 3–5 days)	Wang et al., 2014 [1]
Isobutanol tolerance	*Synechocystis* GT	Constant (not stepwise) 2 g L^{-1} isobutanol	Four independent parallel cultures	Propagated until growth-rate improvement plateaued at ~1000 h; evolved cells collected at 1824 h (~76 days)	100-mL Erlenmeyer flasks, 20 mL BG_{11}	34 °C	40 µmol photons m^{-2} s^{-1}	50 mM $NaHCO_3$ (as the CO_2 source)	Every 3.5 days at OD_{730} 0.03	Matsusako et al., 2017 [40]

Characteristics of the evolved strain	Strain	Regime	Lineages	Duration	Vessel	Temperature	Irradiance	CO_2	Transfer / bottleneck	Reference
**Engineering + ALE**										
PSI-independent photoautotrophic growth	PSI-deficient strain in which all twelve native PSI genes were replaced by their *Arabidopsis* counterparts, with no detectable PSI subunits from either source; two independent parental sublines used as ALE starting material for the first campaign. A freshly constructed Δ*psaAB* deletion strain carrying the reconstructed *fusA*^{I28T} mutation was used as starting material for the second, confirmatory campaign	Campaign 1: single continuous static incubation (glucose withdrawal under very low light), no intermediate transfers, contrasting with classic serial-passage ALE; campaign 2: serial-passage ALE under stepwise glucose restriction and repeated transfers	Campaign 1: twelve parallel replicate cultures (six per parental line), of which eight resumed growth; campaign 2: more than 100 independent replicate cultures, of which three regained the phenotype	Campaign 1: growth resumed after 10–19 weeks (up to 19 weeks total); campaign 2: ~15–16 weeks until phenotype recovery	Campaign 1: 150 mL mixotrophic precultures shifted to sealed, static, foil-covered glucose-free BG_{11}; campaign 2: 50 mL BG_{11} cultures	30 °C (both campaigns)	Campaign 1: ~10 µmol photons m^{-2} s^{-1} (preculture) shifted to 3–5 µmol photons m^{-2} s^{-1} (static incubation); campaign 2: 3–5 µmol photons m^{-2} s^{-1} throughout	Not specified	Campaign 1: no intermediate transfers; single static incubation for up to 19 weeks; campaign 2: serial transfers with a defined bottleneck (resuspension to OD_{720} ~0.1 at each transfer), glucose concentration reduced by 10% per step (0.90 mM down to 0.12 mM)	Ludwiczak et al., 2026 [62]
Enhanced mannitol production under salt stress	ΔCS (*ggpS*/*sps* double-knockout *Synechocystis*, lacking native compatible-solute synthesis), carrying an *mtlD/m1p* mannitol-secretion cassette under a strong constitutive promoter (ΔCS_M); a sister construct under a salt-inducible promoter (ΔCS_IM) was	Constant 200 mM NaCl compared against a stepwise-increasing regime (from 200 to 400 mM in 50 mM increments, advanced once growth rate recovered)	Three replicate ΔCS_M lineages; only one survived to 400 mM; the three "evolved mutants" analyzed (EMP#1-3) are clonal isolates from this single surviving population, not three independent replicates	164th generation reached by the surviving lineage	Triplicate 100 mL shake flasks (20 mL working volume), BG_{11} with 50 mM PIPPS buffer (pH 8), 120 rpm shaking	30 °C	~50 µmol photons m^{-2} s^{-1} red light	Not stated	Diluted from OD_{730} ~2.0 to 0.05 at each transfer (~40-fold dilution); salt concentration advanced by 50 mM once growth rate recovered or stabilized	Wu et al., 2025 [41]

Characteristics of the evolved strain	Strain	Regime	Lineages	Duration	Vessel	Temperature	Irradiance	CO_2	Transfer / bottleneck	Reference
	evolved in parallel but produced far less mannitol and was not carried forward for genomic analysis									

References:

1. Wang, Y.; Shi, M.; Niu, X.; Zhang, X.; Gao, L.; Chen, L.; Wang, J.; Zhang, W. Metabolomic Basis of Laboratory Evolution of Butanol Tolerance in Photosynthetic *Synechocystis* Sp. PCC 6803. *Microb. Cell Fact.* 2014, 13, 1–12, doi:10.1186/s12934-014-0151-y.

40. Matsusako, T.; Toya, Y.; Yoshikawa, K.; Shimizu, H. Identification of Alcohol Stress Tolerance Genes of *Synechocystis* Sp. PCC 6803 Using Adaptive Laboratory Evolution. *Biotechnol. Biofuels* 2017, 10, 1–9, doi:10.1186/s13068-017-0996-5.

41. Wu, W.; Jongbloets, J.A.; Du, W.; Hellingwerf, K.J.; Branco dos Santos, F. Isolation of *Synechocystis* Mutants Overproducing Mannitol Directly from CO_2 via Laboratory Evolution under Increasing Salt Concentration. *ACS Synth. Biol.* 2025, 14, 3557–3567, doi:10.1021/ACSSYNBIO.5C00344.

45. Figueroa-Gonzalez, T.; Chen, W.; Abdel-Salam, E.M.; Štipl, D.; Komenda, J.; Zhivkovikj, M.; Dann, M.; Leister, D. Improving Tolerance to Fluctuating Light through Adaptive Laboratory Evolution in the Cyanobacterium *Synechocystis*. *Nat. Commun.* 2026, 17, 4025, doi:10.1038/s41467-026-72689-x.

48. Dann, M.; Ortiz, E.M.; Thomas, M.; Guljamow, A.; Lehmann, M.; Schaefer, H.; Leister, D. Enhancing Photosynthesis at High Light Levels by Adaptive Laboratory Evolution. *Nat. Plants* 2021, 7, 681–695, doi:10.1038/s41477-021-00904-2.

49. Yoshikawa, K.; Ogawa, K.; Toya, Y.; Akimoto, S.; Matsuda, F.; Shimizu, H. Mutations in Hik26 and Slr1916 Lead to High-Light Stress Tolerance in *Synechocystis* Sp. PCC6803. *Commun. Biol.* 2021, 4, doi:10.1038/S42003-021-01875-Y.

50. Hu, L.; He, J.; Dong, M.; Tang, X.; Jiang, P.; Lei, A.; Wang, J. Divergent Metabolic and Transcriptomic Responses of *Synechocystis* Sp. PCC 6803 to Salt Stress after Adaptive Laboratory Evolution. *Algal Res.* 2020, 47, 101856, doi:10.1016/J.ALGAL.2020.101856.

51. Zhu, X.; Zou, R.; Liu, D.; Liu, J.; Wu, X.; Jiang, J.; Tian, L.; Chen, L.; Sun, T.; Zhang, W. Enhanced Salt Tolerance in *Synechocystis* Sp. PCC 6803 through Adaptive Evolution: Mechanisms and Applications for Environmental Bioremediation. *Microbiol. Res.* 2025, 296, doi:10.1016/j.micres.2025.128140.

52. Xu, C.; Sun, T.; Li, S.; Chen, L.; Zhang, W. Adaptive Laboratory Evolution of Cadmium Tolerance in *Synechocystis* Sp. PCC 6803. *Biotechnol. Biofuels* 2018, 11, 1–15, doi:10.1186/s13068-018-1205-x.

53. Xiong, J.; Dong, Z.; Xie, Y.; Zhang, W.; Sun, T.; Chen, L. Identification of Genes Involved in Cadmium-Ion Tolerance in Evolutionary *Synechocystis* Sp. PCC 6803 Tolerant to Both Cadmium and High Light. *Chin. J. Chem. Eng.* 2024, 74, 74–82, doi:10.1016/J.CJCHE.2024.05.023.

54. Uchiyama, J.; Kanesaki, Y.; Iwata, N.; Asakura, R.; Funamizu, K.; Tasaki, R.; Agatsuma, M.; Tahara, H.; Matsuhashi, A.; Yoshikawa, H.; et al. Genomic Analysis of Parallel-Evolved Cyanobacterium *Synechocystis* Sp. PCC 6803 under Acid Stress. *Photosynth. Res.* 2015, 125, 243–254, doi:10.1007/s11120-015-0111-3.

55. Tillich, U.M.; Wolter, N.; Franke, P.; Dühring, U.; Frohme, M. Screening and Genetic Characterization of Thermo-Tolerant *Synechocystis* Sp. PCC6803 Strains Created by Adaptive Evolution. *BMC Biotechnol.* 2014, 14, doi:10.1186/1472-6750-14-66.

56. Wang, W.; Pan, K.; Gao, J.; Chen, L.; Sun, T.; Zhang, W. Mutations in AtpB and LexA Confer Temperature Tolerance in an Evolved *Synechocystis* Sp. PCC 6803 Strain Resistant to High Temperature and Light. *Plant Physiol. Biochem.* 2026, 235, doi:10.1016/j.plaphy.2026.111384.

57. Hou, G.; Liu, Y.; Liu, W.; Zhang, M.; Ding, H.; Zhou, W. Tolerance Evolution in Cyanobacteria under Chronic Copper and Moxifloxacin Stress: Phenotypic Plasticity and Genomic Fixation. *Algal Res.* 2025, 91, 104327, doi:10.1016/J.ALGAL.2025.104327.

58. Wu, P.; Wei, K.; Hu, T.; Chen, J.; Luan, G.; Sun, L.; Fan, J. Genome-Wide Hypermutation-Engineered *Synechocystis* Sp. PCC 6803 Reveals Membrane-Mediated Triclosan Resistance. *Plant Physiol.* 2026, 200, doi:10.1093/PLPHYS/KIAG121.

59. Hu, L.; Fang, X.; Wen, L.; Zhang, H.; Peng, B.; Li, C. Molecular Insights into the Enhanced Growth of Cyanobacteria by Adaptive Laboratory Evolution in Wastewater Environments. *Algal Res.* 2024, 83, 103724, doi:10.1016/j.algal.2024.103724.

60. Kukil, K.; Englund, E.; Crang, N.; Hudson, E.P.; Lindberg, P. Laboratory Evolution of *Synechocystis* Sp. PCC 6803 for Phenylpropanoid Production. *Metab. Eng.* 2023, 79, 27–37, doi:10.1016/J.YMBEN.2023.06.014.

61. Hosoda, K.; Habuchi, M.; Suzuki, S.; Miyazaki, M.; Takikawa, G.; Sakurai, T.; Kashiwagi, A.; Sueyoshi, M.; Matsumoto, Y.; Kiuchi, A.; et al. Adaptation of a Cyanobacterium to a Biochemically Rich Environment in Experimental Evolution as an Initial Step toward a Chloroplast-Like State. *PLoS One* 2014, 9, e98337, doi:10.1371/JOURNAL.PONE.0098337.

62. Ludwiczak, M.; Dann, M.; Figueroa-Gonzalez, T.; Abdel-Salam, E.M.; Chen, W.; Schwenkert, S.; Lehmann, M.; Zhivkovikj, M.; Noureddine, M.; Linhartová, M.; et al. Photosystem I-Independent Oxygenic Photosynthesis in Cyanobacteria. *Nat. Commun.* 2026, 17, doi:10.1038/S41467-026-74903-2.
