# Supplementary material for: Adaptive Laboratory Evolution in Synechocystis sp. PCC 6803: Current Status and Perspectives
Source: Microorganisms. 2026 Aug 19;14(8):1836. doi: 10.3390/microorganisms14081836 (PMC13515095; doi:10.3390/microorganisms14081836)
Supplement: Supplementary file 1 [file microorganisms-14-01836-s001.zip › Supplementary Table S3.pdf]

Supplementary Table S3. Genomic outcomes and functional validation of the ALE studies included in **Table 1**. Abbreviations: ALE, adaptive laboratory evolution; WT, wild type; GT, glucose tolerant; LT, laboratory-adapted strain; U, one round of UV mutagenesis; M, one round of methyl methanesulfonate (MMS) mutagenesis; lineage names (e.g., UM, UMU, UMMM) indicate the sequential order of UV (U) and MMS (M) mutagenesis rounds; Tol, high light tolerant strain; FL0, non-lethal fluctuating-light regime; FL+, lethal fluctuating-light regime; HL, high light; LL, low light; SNP, single-nucleotide polymorphism; PCA, principal component analysis; WGCNA, weighted gene co-expression network analysis; DEGs, differentially expressed genes; ROS, reactive oxygen species; LED, light-emitting diode.

Characteristics of the evolved strain	Mutations	Reference genome	Sequencing	Reverse engineering	Stability	Cross-tolerance	Trade-offs	Endpoint phenotype	Reference
**Environmental stressors**									
High light tolerance	More than 100 mutations (SNPs and indels), grouped into five haplotypes	The ancestor ($LT_{t=0}$) was resequenced to serve as the reference genome	Population-level sequencing (13 intermediate and 7 final batch-culture metagenomes) plus clone-level sequencing (28 monoclonal isolates)	Two representative mutations, NdhF1-F124L (gene *ndhF1*) and EF-G2-R461C (gene *fusB*), individually reintroduced into LT and confirmed as causal	Not reported	Not tested	Reduced growth/dry mass under low light (50 µmol)	Growth was visually retained at 3000 µmol photons m^{-2} s^{-1}, while quantitative dry-mass accumulation was assessed up to 2000 µmol photons m^{-2} s^{-1}	Dann et al., 2021 [48]
High light tolerance	Two non-synonymous mutations in *hik26* and *slr1916*	*Synechocystis* GT-I reference genome (NC_017038.1) used for comparison	Whole-genome sequencing of parental PCC6803 and Tol; four single-colony Tol isolates subsequently characterized	Both *hik26* and *slr1916* functionally validated by deletion and introduction of evolved alleles	Phenotype remained stable after 15 days (5 subcultures) under low light	Not tested	No low-light growth defect detected; Tol showed growth comparable to WT at 40 µmol photons m^{-2} s^{-1}	The Tol strain showed no growth inhibition at 9000 µmol photons m^{-2} s^{-1}, whereas growth of the WT almost stopped	Yoshikawa et al., 2021 [49]
Fluctuating-light tolerance	412 mutations absent from both controls; of 101 fully segregated alleles, 44 unique to evolved strains (28 affecting proteins/structural RNAs). *sll0518* and *pam68* mutated in all 24 strains; *rpaB* was independently hit by two mutations (T183P in 3 isolates, D194G in 3 other), indicating convergent evolution at the same locus	Same LT background and reference genome assembly (ASM972v1, $LT_{t=0}$) used as the reference genome	Whole-genome resequencing of all 24 monoclonal isolates (clone-level), plus LT and the original motile WT as controls.	*sll0518*$_{A133V}$, *pam68*$_{S113G}$, and *rpaB*$_{T183P}$ individually reconstructed in the LT background by marker-less recombination and functionally validated	Not tested	*rpaB*$_{T183P}$ conferred tolerance to both the lethal FL+ regime and constant high light; *sll0518* and *pam68* enhanced tolerance to the non-lethal FL0 regime only	*rpaB*$_{T183P}$ reduced growth under constant low light.	Evolved isolates survived and grew under a fluctuating-light regime that was lethal to the ancestral strain	Figueroa-Gonzalez et al., 2026 [45]

Characteristics of the evolved strain	Mutations	Reference genome	Sequencing	Reverse engineering	Stability	Cross-tolerance	Trade-offs	Endpoint phenotype	Reference
Salt tolerance	No mutations identified; no DNA sequencing was performed	Neither the ancestral nor the evolved strains were whole genome resequenced	No genomic (DNA) sequencing; phenotypes characterized exclusively at the metabolomic (58 metabolites) and transcriptomic level	No reverse-genetic validation; differentially expressed candidate genes (e.g., potassium transporters) were identified but not functionally validated, as no reverse-genetic testing was performed	Not reported	Not reported	Not reported	Eight of the ten populations yielded salt-tolerant strains at 3% NaCl, with divergent metabolic and transcriptomic subclusters identified among the evolved lines	Hu et al., 2020 [50]
Salt tolerance	8 mutated loci relative to wild type: 3 SNPs (*sll1867*, *slr1670*, *sll0689*) and 5 indels (*slr1753*, *sll1755*, *sll2011*, *sll0377*, *sll0762*)	Not separately described; comparisons were made directly against a sequenced wild type (see Genomic outcome)	Whole-genome resequencing (NCBI BioProject PRJNA1187691) of single colonies from the four isolates	Knockout and complementation performed for all eight genes in wild type; overexpression was attempted for all eight but could not be obtained for *sll0689;* only two showed a clear causal, adaptive effect: knockout of *slr1670* alone improved salt tolerance and increased intracellular glucosylglycerol by 266.3%; overexpression of the evolved *slr1753* allele (not the gene generally) improved growth specifically at 5.0% NaCl. *sll0689* was essential for baseline growth; three further genes (*sll2011*, *sll0377*, *sll0762*) impaired growth when knocked out but were not otherwise validated; complementation restored wild-type-like growth for all eight genes but did not reproduce the evolved strains' enhanced tolerance; combining the *slr1670* knockout with *slr1753* (evolved)-overexpression caused growth inhibition (negative epistasis)	Not directly tested for reversion; a representative evolved isolate (ALE-3) showed morphology, pigment absorption, and chlorophyll-fluorescence parameters comparable to wild type under standard, salt-free conditions	Not tested; the evolved strain was instead applied to seawater desalination and saline-soil bioremediation	None detected under standard, salt-free conditions (see Stability)	Evolved strains grew at 6.5% NaCl (versus a 4.0% threshold in the parental strain); the representative isolate ALE-3 accumulated 82.7% more intracellular glucosylglycerol than wild type under 5.0% NaCl stress	Zhu et al., 2025 [51]
Cadmium tolerance	Nine confirmed mutations (6 non-synonymous SNPs, 1	WT was resequenced alongside ALE-9.0; both were compared against the published NCBI reference genome (NC_000911), not	Whole-genome resequencing of both WT and ALE-9.0 (clone-level); candidate	Knockout of all nine loci in both WT and ALE-9.0 backgrounds; overexpression of WT and evolved allele	Not tested	Stressor-specific (improved for Zn^{2+}, Co^{2+}, and high light; not for Cu^{2+}, ethanol, or	Slightly reduced growth with a pigment shift (less phycocyanin, more carotenoid) under	Evolved strains grew at 9.0 μM $CdSO_4$, a 95% improvement over the WT's 4.6 μM threshold	Xu et al., 2018 [52]

Characteristics of the evolved strain	Mutations	Reference genome	Sequencing	Reverse engineering	Stability	Cross-tolerance	Trade-offs	Endpoint phenotype	Reference
	synonymous SNP in *sll1586*, 1 structural variant [659-bp deletion truncating *slr0454*], 1 insertion in *slr1753*), out of an initial 14 candidate variants	against a custom WT-derived reference	variants validated by Sanger sequencing; the intermediate isolate ALE-7.0 was also assessed by targeted Sanger sequencing at the 9 confirmed loci to trace mutation timing	variants for all nine genes in the WT background; only *slr0454* causally validated by gain-of-function (evolved truncated allele improved Cd^{2+} tolerance more than WT allele); three genes (*slr0623, slr0721, slr0798*) supported by both knockout and overexpression, though overexpression did not distinguish the evolved allele from the WT allele (gene-dosage effect); all three also transcriptionally upregulated under Cd^{2+} stress in ALE-9.0; three genes (*slr0774, slr1753, ssr1480*) supported by knockout, though overexpression had an inhibitory effect for all three; *ssr1480*'s knockout phenotype was specific to the WT background (not replicated in ALE-9.0), unlike *slr0774* (both backgrounds); *slr1302* not assessable (knockout impairs baseline growth independent of Cd^{2+}). *sll1586* showed no knockout phenotype in either background and no overexpression effect (the only locus with no functional support at all)		H_2O_2 (latter two showed decreased tolerance))	standard, cadmium-free conditions		
Cadmium and high light tolerance	28 total mutations (18 intragenic: 2 synonymous SNPs, 13 nonsynonymous SNPs, 3 indels, spanning 15 genes; 10 intergenic)	Not resequenced as a custom reference; both the parent strain (HL-0) and evolved isolates were compared against the published NCBI reference genome (NC_000911)	Whole-genome resequencing (Illumina HiSeq/NovaSeq, 2×150 bp) of single colonies from HL-0 and the three evolved isolates (clone-level); candidate	12 of the 15 mutated genes selected for single-gene knockout in HL-0 by chloramphenicol-cassette homologous recombination; knockout could not be obtained for *slr0798* and *slr1718,* leaving 10	Not tested for reversion under selection-free conditions	Not tested (no independent, unrelated stressor examined; the study specifically evaluates combined Cd^{2+} + high light tolerance, not	None detected: in Cd^{2+}-free (but still high light) medium, growth rates of the evolved isolates HLHC-1, HLHC-2, and HLHC-3 were comparable to HL-0; the representative isolate HLHC-3 also showed	Evolved isolates tolerated 5.5 mM Cd^{2+} under constant high light (600 µmol photons m^{-2} s^{-1}), an ~83% improvement over the 3.0 mM threshold of the high-light-adapted parental strain HL-0;	Xiong et al., 2024 [53]

Characteristics of the evolved strain	Mutations	Reference genome	Sequencing	Reverse engineering	Stability	Cross-tolerance	Trade-offs	Endpoint phenotype	Reference
	identified across the three evolved isolates relative to HL-0. Table 1 lists 12 genes (13 mutation events, since *sll0698* was independently mutated at different sites in HLHC-1 and HLHC-2) after excluding synonymous and unannotated-protein mutations; all are nonsynonymous SNPs except a 25-bp frameshift insertion in *slr1718* (HLHC-2)		mutations validated by Sanger sequencing	successful knockouts, each subsequently complemented with the evolved allele under the strong Pcpc560 promoter. Four genes functionally implicated: *ssl2615* and *sll1732* knockouts were more Cd^{2+}-sensitive than HL-0, with complementation restoring growth to approximately the HL-0 level. *ssr1480* knockout showed a confounding baseline growth defect independent of Cd^{2+}, but its complementation exceeded HL-0 growth under Cd^{2+} stress (gain-of-function). *sll1659* showed the opposite pattern (knockout grew slightly better than HL-0 under Cd^{2+}, while complementation grew worse) indicating a negative regulatory role rather than a positive contribution; two further genes (*slr0623*, *sll0030*) showed knockout sensitivity to Cd^{2+}, but complementation failed to restore HL-0-level growth (*sll0030* complementation was even worse than its own knockout), leaving their role inconclusive; the remaining four knockout strains showed no distinct Cd^{2+}-tolerance phenotype		tolerance to a third stressor)	no significant morphological difference from HL-0 by SEM under Cd^{2+}-free conditions	under Cd^{2+} stress, the representative isolate HLHC-3 showed higher absorption at 450–525 nm (more carotenoids) than HL-0 and resisted the cell aggregation/lysis seen in HL-0 (SEM); metabolomic analysis showed elevated ATP and depleted upper-glycolysis intermediates relative to HL-0 under Cd^{2+} stress	
Acid tolerance	8 mutations in A.A.1, 9 in A.A.2, relative to the parent strain (11 distinct genes/positions total); two	Resequenced as the baseline for calling adapted-strain mutations	Whole-genome resequencing; not specified whether performed on a bulk population or an isolated single-colony clone	None performed for any SNP gene; only correlational qRT-PCR expression analysis, which found no consistent expression difference between parent and adapted	Not tested	Stressor-specific, no improvement to salt stress (0.5 M NaCl); confirmed improvement in tolerance to non-ionic hyperosmotic	Confirmed. Adapted strains grew more poorly than the parent strain at the ancestral, non-selective pH (8.0), and showed ~20% smaller average cell diameter than the	Evolved strains grew at pH 5.5, a condition at which the parental strain was arrested	Uchiyama et al., 2015 [54]

Characteristics of the evolved strain	Mutations	Reference genome	Sequencing	Reverse engineering	Stability	Cross-tolerance	Trade-offs	Endpoint phenotype	Reference
	mutations (*sll1951*, *sll0914*) shared by both lineages; F_oF_1-ATPase operon hit in both lineages but at different genes (*sll1321* in A.A.1, *sll1322* in A.A.2); convergent evolution at the same complex			strains for most SNP genes		stress (0.5 M sorbitol)	parent under non-stress conditions (cell shape unchanged)		
High temperature tolerance	SNPs and indels in eight genes, *clpC* (mutated independently at five distinct positions), *pnp* (two positions), *pyk2*, *sigF*, *nlpD*, *pilJ*, *cya1*, and *pyrR*	Resequenced at 19-fold coverage as the baseline	Population-level sequencing of the mutIV-mix-2 metagenomes (up to 123.5-fold coverage) plus clone-level sequencing of the five monoclonal strains (18–32-fold coverage)	None performed; convergence of independent mutations across lineages used as indirect evidence instead; *pyk2* excluded from functional testing as an essential glycolytic gene	Not tested	Not tested	Most individual mix-derived strains failed to outperform the wild type alone, suggesting fitness costs or context-dependencies (discussed but not quantified)	The most evolved strain mixture (mutIV-mix-2) grew stably at 45.8 °C in the photobioreactor, a 0.8 °C improvement over the original mutIV-mix (45.0 °C); individually validated monoclonal strains reached between 44.9 and 45.1 °C	Tillich et al., 2014 [55]
High temperature and high light tolerance	21 mutations relative to wild type identified; after subtracting mutations already present in the HL-4 parent, 6 mutations across 5 genes attributed to this second round of evolution, *sll1626*/l*exA* (insertion, all 3 strains, plus a further SNP in HLHT-2), *sll1457*/*rfbJ* (HLHT-2), *sll1531* (HLHT-2), *sll1326*/*atpA* (HLHT-1 and HLHT-3), *slr1329/atpB* (HLHT-2)	WT was independently resequenced in this study; reads were aligned against the published CyanoBase reference genome; mutations already present in the previously-evolved HL-4 parent (the actual ALE starting strain) were subtracted to isolate the 6 mutations specific to this HT/HL evolution round	Whole-genome resequencing of the three evolved strains and wild type; HL-4-parent mutations subtracted, confirmed by Sanger sequencing	Knockout (not achievable for *sll1626*, suggesting essentiality), CRISPR-interference knockdown, and overexpression of all five candidate genes in wild type, plus allele replacement/overexpres sion in the HL-4 background; *slr1329* (AtpB) showed the strongest causal evidence across the assays; *sll1457* (RfbJ) showed a partial, inconsistent signal in HL-4-background replacement (modest improvement in liquid-culture heat-shock recovery, but no colony formation under the more stringent plate assay); the authors concluded it does not	Not reported	Not reported	Not reported	Evolved strains showed lower ROS accumulation, improved photosynthetic performance and pigment retention, and greater dry weight and glycogen content than the wild type under combined 40 °C / 750 µmol photons m^{-2} s^{-1} conditions	Wang et al., 2026 [56]

Characteristics of the evolved strain	Mutations	Reference genome	Sequencing	Reverse engineering	Stability	Cross-tolerance	Trade-offs	Endpoint phenotype	Reference
				confer tolerance alone, similarly to *sll1626*.; *sll1626* (LexA), despite being the most convergently mutated locus, was not causally validated; *sll1326* (AtpA) and *sll1531* were tested by knockout, CRISPR-interference knockdown, and overexpression in WT, with no gain-of-function benefit observed; remaining candidate associations were not causally validated					
Copper and moxifloxacin tolerance	Three classes of variant: (1) stressor-specific SNPs in rpoB (G424S under Cu^{2+}, A997V under moxifloxacin, both near but outside the core rifampicin-resistance-determining region); (2) large-scale tandem duplications spanning megabase regions of the chromosome and plasmids pSYSM/pSYSG (and, for Cu2+, pSYSA); (3) copy-number gains of plasmids pSYSA and pSYSX, plus a shared chromosomal deletion reducing expression of amino-acid,	NCBI reference genome GCF_000009725.1 used for resequencing alignment	Whole-genome resequencing (population-level, DNBSEQ platform, 382x mean depth, 100% genome coverage); transcriptomics also performed (614 DEGs under Cu^{2+}, 1163 under moxifloxacin)	None performed; causal attribution rests on a correlational multi-omics argument plus an explicit stability test rather than genetic validation	Explicit 30-day stressor-removal test; tolerance to the selecting agent persisted	Substantially reverted to non-selecting antibiotics after the 30-day stressor-removal period	Modest, delayed-onset fitness cost. Cu^{2+}-adapted populations grew slightly but significantly slower than controls by day 16 under standard conditions; no cost apparent over shorter timescales or under active stress (adapted populations instead showed 1.24–4.03-fold higher biomass than unadapted controls)	Populations developed stable tolerance to the selecting agents (copper and moxifloxacin) and transient cross-tolerance to several additional antibiotics, with the two phenotypes associated with distinct point-mutation versus transcriptional/structural responses	Hou et al., 2025 [57]

Characteristics of the evolved strain	Mutations	Reference genome	Sequencing	Reverse engineering	Stability	Cross-tolerance	Trade-offs	Endpoint phenotype	Reference
	purine-biosynthesis, and DNA-repair (*recA*) genes								
Triclosan tolerance	62 total mutations (46 SNPs: 15 nonsynonymous /19 synonymous coding + 12 intergenic; 16 indels: 13 nonsynonymous coding + 3 noncoding) identified across 9 TCS-tolerant strains (3 ALE-derived isolates from the ED30 lineage, plus 3 randomly picked colonies each from complemented hypermutator lines R-HM24 and R-HM33) relative to WT; two mutations recurred across strains: *fabI* (Syn_*slr1051*, C504229T/A116V) in 100% of strains, and an uncharacterized gene (Syn_*sll1969)* in 66.7%, co-occurring in 3 isolates. ALE-derived isolates carried substantially more missense mutations on average (23.33) than hypermutator-derived isolates (4.67–6.00), reflecting greater genetic background	External, published NCBI reference genome (NC_000911 / GCF_000009725.1) used for alignment; a WT control strain was also resequenced in parallel, and its mutations were subtracted to filter out sequencing errors and background variants	Whole-genome resequencing (NovaSeq 6000; BWA-MEM alignment, GATK4 variant calling, ANNOVAR annotation), validated by Sanger sequencing; clone-level for the hypermutator-derived isolates (3 randomly selected colonies per strain). The sequencing basis (bulk vs. clonal) for the 3 ALE-derived isolates was not explicitly specified	Both recurrent candidate mutations individually introduced into WT via homologous recombination. *fabI* (A116V) reconstruction alone conferred enhanced TCS tolerance (strain Mut-*fabI*), confirming causality. Syn_*sll1969* reconstruction alone did not significantly enhance tolerance despite its high recurrence frequency, indicating it is not independently sufficient. Separately, CRISPR-Cpf1 editing restored native DNA-repair gene function (*mutS* in R-HM24; *mutS*+*mutM* in R-HM33) to revert the hypermutator phenotype before phenotyping	Not tested	Confirmed: TCS-tolerant strains showed reduced sensitivity to several non-selecting antibiotics; Hypermutator-hderived strains (HM24, HM33) showed broad cross-resistance to aminoglycosides (kanamycin, spectinomycin), linked to missense mutations in ABC-transporter genes Syn_Slr0559 and Syn_Slr1209; the ALE-derived strain (ED30) showed narrower cross-tolerance, limited to kanamycin (54.00% inhibition at 4×EC50 vs. 89.78% for WT), linked to a mutation in the histidine-kinase gene Syn_*sll1353*	Not directly quantified for ED30 or the hypermutator strains under standard, TCS-free conditions. ED30 carries additional mutations in Syn_*sll1502* (glutamate synthase) and Syn_*slr2094* (fructose-1,6-bisphosphatase), speculated to impose metabolic imbalance limiting its resistance capacity relative to the hypermutator strains; the reconstructed single-mutation strain Mut-*fabI* grew comparably to WT under standard conditions	ED30 (ALE-derived): 96 h EC50 of 21.59 µg/L TCS, a 1.40-fold improvement over WT (15.41 µg/L); hypermutator-complemented strains R-HM24 and R-HM33: EC50 of 4.963 and 5.238 mg/L — 322- and 340-fold improvements over WT, respectively, exceeding the highest previously reported tolerance in the eukaryotic microalga *Chlamydomonas reinhardtii*. R-HM24 and R-HM33 also showed enhanced TCS biodegradation (7-day removal of 57.19% and 48.50% at 10 mg/L TCS, vs. 32.34% for WT) while maintaining robust growth	Wu et al., 2026 [58]

Characteristics of the evolved strain	Mutations	Reference genome	Sequencing	Reverse engineering	Stability	Cross-tolerance	Trade-offs	Endpoint phenotype	Reference
	noise from conventional serial-passage evolution								
Adaptation to wastewater cultivation	Not identified. No whole-genome resequencing or any DNA-level mutation analysis was performed in this study	Not applicable (no genomic resequencing performed)	Transcriptomic only: RNA-seq (Illumina HiSeq 4000; TruSeq Stranded Total RNA Library Prep) comparing the evolved strain (WW) vs. WT, validated by qRT-PCR for 10 selected genes (significant correlation between RNA-seq and qRT-PCR fold changes, r = 0.830); no genomic (DNA) sequencing was performed	None performed. No knockout, complementation, overexpression, or allele-reconstruction experiments; findings rest on correlational transcriptomic (165 DEGs, 27 photosynthesis-related genes) and physiological (photosynthetic O_2 evolution) evidence only	Not tested	Not tested	Not directly tested (no comparison of WW vs. WT growth in standard BG_{11} medium); a related but distinct observation: pre-culturing WW in BG_{11} before wastewater inoculation slightly impaired its subsequent growth in wastewater relative to WW inoculated directly from wastewater-adapted stock	WW reached a higher final OD_{730} (2.25 vs. 1.61) and dry weight (0.693 vs. 0.526 g L^{-1}) than WT by day 13, with a significantly higher specific growth rate (0.317 vs. 0.289 day^{-1}). WW showed numerically (but not statistically significantly) higher nutrient-removal efficiency than WT (total nitrogen: 35.55% vs. 34.00%; total phosphorus: 60.95% vs. 59.05%); lipid content was similar between strains (16.19% WW vs. 14.95% WT, not significant), but WW had significantly higher polyunsaturated fatty acid content (25.1% vs. 20.47%), which significantly reduced oxidation stability (7.40 vs. 8.46 h) and saponification number (201.97 vs. 202.75) relative to WT, while both strains remained within relevant biodiesel-quality standards (ASTM D6751/EN14214)	Hu et al., 2024 [59]
**Tolerance to products**									
Phenylalanine tolerance	4 mutations shared by all 3 fully sequenced PRMs and absent from wild type, *ccmA* (DAHP	External reference: *Synechocystis* GT-Kazusa (NC_000911); wild type also independently re-sequenced to distinguish pre-existing from PRM-specific mutations	Whole-genome sequencing (Illumina NextSeq, clone-level) of 3 PRMs referenced against the	None performed for *ccmA* or any other candidate gene. Proposed mechanism (loss of Phe feedback inhibition of DAHP synthase) inferred from	Phenotype re-confirmed stable upon retesting (no growth inhibition at 2 mM Phe,	Not directly applicable (production/tolerance-coupled trait, not tested against other stressors)	In most PRM backgrounds, downstream phenylpropanoid (tCA/pCou) productivity after PAL/TAL	Evolved strains grew at 12 mM phenylalanine; PRM8 secreted over 600 mg L^{-1} into the medium, and its PAL/TAL-	Kukil et al., 2023 [60]

Characteristics of the evolved strain	Mutations	Reference genome	Sequencing	Reverse engineering	Stability	Cross-tolerance	Trade-offs	Endpoint phenotype	Reference
	synthase, always within its N-terminal ferredoxin-like regulatory domain, differing between strains), *slr1916* (Pro42Leu), *sll1995* (frameshift/early stop), and one shared non-coding SNP; all 10 PRMs carry some mutation within the same *ccmA* regulatory domain, a strong convergent-evolution signal		external genome and the re-sequenced wild type; targeted amplification/Sanger sequencing of all three candidate loci (*ccmA*, *slr1916*, *sll1995*) in the remaining 7 PRMs	structural homology to a characterized bacterial DAHP synthase, not experimentally validated in *Synechocystis*	unlike wild type); no formal reversion/stressor-removal test performed		overexpression was lower than in the wild-type background; the best-performing isolate (PRM8) was an exception, outperforming the wild-type background under high-density cultivation	engineered derivatives produced over 1 g L^{-1} of trans-cinnamic acid or ***p***-coumaric acid in high-density cultivation within four days.	
Toxic amino acids tolerance	*ccmA* and other loci (including BgtB and a penicillin-binding protein, per the main text)	NCBI reference sequence NC_000911.1 used for read mapping; the ancestral strain was itself resequenced (~302-fold coverage), revealing 20 pre-existing SNPs and 8 indels relative to the reference, also present in later samples	Population-level sequencing of bulk culture at three time points (ancestral, initially adaptive, and evolved), rather than single-colony isolation	None reported (association-level evidence only)	Not reported	Not reported	None detected; no decrease in specific growth rate observed in any tested medium, including the inorganic medium BG_{11} (autotrophic/photosynthetic capacity preserved)	The evolved population reached, in TCM1 (containing all six toxic amino acids at ~1 mM each), a growth rate matching that of the non-toxic control	Hosoda et al., 2014 [61]
n-Butanol tolerance	None identified, no DNA sequencing was performed	Not stated (no resequencing reported)	None; metabolomics-only study (GC-MS/LC-MS and WGCNA analysis across time-course samples S0–S4)	Not possible, no candidate genes identified	Not reported	Not reported	Not reported	Butanol tolerance increased by 150% (from 0.2% to 0.5% v/v)	Wang et al., 2014 [1]
Isobutanol tolerance	SNPs and indels in *mcpA*, *envD*, and *hik43* (mostly loss-of-function frameshifts/stop codons); T(2) additionally carries 8 extra passenger mutations linked to a stop-codon mutation in *mutS* (mismatch repair),	External reference: *Synechocystis* GT-I strain (NCBI NC_017038.1); parental strain SNPs relative to this reference were also identified (SAMtools) to distinguish pre-existing from newly acquired mutations in evolved isolates	MiSeq paired-end resequencing at the clone level (single-colony isolates T(1)–T(4))	Single and double knockouts (*ΔmcpA*, *Δhik43*, *ΔenvD*, *ΔmcpA*/*ΔenvD*, *Δhik43*/*ΔenvD*) in the parental background confirmed the causal contribution and synergistic effects of these loci; exact evolved mutations were reconstructed for *mcpA* and *envD* (not *hik43*). Introducing an ethanol-production pathway	Not tested	Extended to ethanol, n-butanol, and isopentanol	Slight growth reduction of the Δ*envD* single mutant at 2 g L^{-1} isobutanol	Evolved strains grew at 5 g L^{-1} isobutanol, versus complete growth inhibition of the parental strain at that concentration (parental growth already reduced ~50% at 2 g L^{-1})	Matsusako et al., 2017 [40]

Characteristics of the evolved strain	Mutations	Reference genome	Sequencing	Reverse engineering	Stability	Cross-tolerance	Trade-offs	Endpoint phenotype	Reference
	indicating a hypermutator phenotype			into the T(1) background demonstrated a direct production benefit (142% of control productivity)					
**Engineering + ALE**									
PSI-independent photoautotrophic growth	Recurrent *fusA* mutations (nine alleles, in 7 of 8 lines) and a shared *lmbP* mutation (in the remaining line and three others)	External reference: *Synechocystis* genome (ASM972v1, NCBI/Kazusa); both parental sublines and wild type also independently re-sequenced to subtract background/pre-existing mutations	Whole-genome sequencing (population/culture-level, not confirmed clonal) of the 8 evolved lines from the first ALE campaign plus both parental sublines and wild type; sequencing status of the second campaign's 3 evolved lines not specified in the primary text	*fusA* alleles individually reintroduced into the parental background and confirmed causal. Functional dissection: *ndhB* knockout abolished the evolved phenotype (complete segregation could not be achieved); *pgr5* knockout did not abolish the phenotype, though it reduced the growth rate	Not explicitly tested for reversion	Not applicable (capability-gain rather than stress-tolerance study)	Reduced growth rate	Evolved strains resumed photoautotrophic growth, oxygen evolution, and ^{13}C-CO_2 fixation despite the complete absence of Photosystem I	Ludwiczak et al., 2026 [62]
Enhanced mannitol production under salt stress	Initially 1001 candidate mutations across 85 genes; narrowed by PCA/enrichment analysis to 4 mutations in 2 genes (3 confirmed by Sanger sequencing). Three distinct *pnp* alleles (27 bp deletion shared by the evolved isolates 1 and 3, an unconfirmed insertion in evolved isolate 1, G584S unique to evolved isolate 2), plus a R188Q substitution in *sigA/rpoD1* (evolved isolate 2 only)	Custom-modified external reference: *Synechocystis* sp. GT-S assembly (NCBI GCF_000270265.1/ASM27026v1), adjusted to include the parental (OMP) strain's engineered mannitol-cassette sequence	Whole-genome resequencing (breseq, polymorphism mode, accounting for polyploidy)	Partial disruption of *pnp* in the non-evolved parental producer (complete knockout not achievable, consistent with *pnp* being essential) increased mannitol production 6.4-fold; this accounted for only a fraction of the ~24-fold increase in the true evolved isolates; no complementation experiment performed, so *pnp*'s role is supported but not fully validated	Not tested	Not tested	Partial-*pnp*-knockout strain, ~30% slower growth and reduced salt tolerance (surviving only to 250 mM, versus ~300 mM for the non-evolved parental strain producer and up to 450 mM for the fully evolved strains)	Evolved isolates produced 27.7 mg mannitol L^{-1} OD_{730}^{-1} at 350 mM NaCl (~24-fold over the parental strain), accumulating ~700 mg L^{-1} total mannitol over 14 days versus ~45 mg L^{-1} for the parental strain, with maximum tolerated salinity increasing from 300 to 450 mM	Wu et al., 2025 [41]

References:

1. Wang, Y.; Shi, M.; Niu, X.; Zhang, X.; Gao, L.; Chen, L.; Wang, J.; Zhang, W. Metabolomic Basis of Laboratory Evolution of Butanol Tolerance in Photosynthetic *Synechocystis* Sp. PCC 6803. *Microb. Cell Fact.* 2014, 13, 1–12, doi:10.1186/s12934-014-0151-y.

40. Matsusako, T.; Toya, Y.; Yoshikawa, K.; Shimizu, H. Identification of Alcohol Stress Tolerance Genes of *Synechocystis* Sp. PCC 6803 Using Adaptive Laboratory Evolution. *Biotechnol. Biofuels* 2017, 10, 1–9, doi:10.1186/s13068-017-0996-5.

41. Wu, W.; Jongbloets, J.A.; Du, W.; Hellingwerf, K.J.; Branco dos Santos, F. Isolation of *Synechocystis* Mutants Overproducing Mannitol Directly from CO_2 via Laboratory Evolution under Increasing Salt Concentration. *ACS Synth. Biol.* 2025, 14, 3557–3567, doi:10.1021/ACSSYNBIO.5C00344.

45. Figueroa-Gonzalez, T.; Chen, W.; Abdel-Salam, E.M.; Štipl, D.; Komenda, J.; Zhivkovikj, M.; Dann, M.; Leister, D. Improving Tolerance to Fluctuating Light through Adaptive Laboratory Evolution in the Cyanobacterium *Synechocystis*. *Nat. Commun.* 2026, 17, 4025, doi:10.1038/s41467-026-72689-x.

48. Dann, M.; Ortiz, E.M.; Thomas, M.; Guljamow, A.; Lehmann, M.; Schaefer, H.; Leister, D. Enhancing Photosynthesis at High Light Levels by Adaptive Laboratory Evolution. *Nat. Plants* 2021, 7, 681–695, doi:10.1038/s41477-021-00904-2.

49. Yoshikawa, K.; Ogawa, K.; Toya, Y.; Akimoto, S.; Matsuda, F.; Shimizu, H. Mutations in Hik26 and Slr1916 Lead to High-Light Stress Tolerance in *Synechocystis* Sp. PCC6803. *Commun. Biol.* 2021, 4, doi:10.1038/S42003-021-01875-Y.

50. Hu, L.; He, J.; Dong, M.; Tang, X.; Jiang, P.; Lei, A.; Wang, J. Divergent Metabolic and Transcriptomic Responses of *Synechocystis* Sp. PCC 6803 to Salt Stress after Adaptive Laboratory Evolution. *Algal Res.* 2020, 47, 101856, doi:10.1016/J.ALGAL.2020.101856.

51. Zhu, X.; Zou, R.; Liu, D.; Liu, J.; Wu, X.; Jiang, J.; Tian, L.; Chen, L.; Sun, T.; Zhang, W. Enhanced Salt Tolerance in *Synechocystis* Sp. PCC 6803 through Adaptive Evolution: Mechanisms and Applications for Environmental Bioremediation. *Microbiol. Res.* 2025, 296, doi:10.1016/j.micres.2025.128140.

52. Xu, C.; Sun, T.; Li, S.; Chen, L.; Zhang, W. Adaptive Laboratory Evolution of Cadmium Tolerance in *Synechocystis* Sp. PCC 6803. *Biotechnol. Biofuels* 2018, 11, 1–15, doi:10.1186/s13068-018-1205-x.

53. Xiong, J.; Dong, Z.; Xie, Y.; Zhang, W.; Sun, T.; Chen, L. Identification of Genes Involved in Cadmium-Ion Tolerance in Evolutionary *Synechocystis* Sp. PCC 6803 Tolerant to Both Cadmium and High Light. *Chin. J. Chem. Eng.* 2024, 74, 74–82, doi:10.1016/J.CJCHE.2024.05.023.

54. Uchiyama, J.; Kanesaki, Y.; Iwata, N.; Asakura, R.; Funamizu, K.; Tasaki, R.; Agatsuma, M.; Tahara, H.; Matsuhashi, A.; Yoshikawa, H.; et al. Genomic Analysis of Parallel-Evolved Cyanobacterium *Synechocystis* Sp. PCC 6803 under Acid Stress. *Photosynth. Res.* 2015, 125, 243–254, doi:10.1007/s11120-015-0111-3.

55. Tillich, U.M.; Wolter, N.; Franke, P.; Dühring, U.; Frohme, M. Screening and Genetic Characterization of Thermo-Tolerant *Synechocystis* Sp. PCC6803 Strains Created by Adaptive Evolution. *BMC Biotechnol.* 2014, 14, doi:10.1186/1472-6750-14-66.

56. Wang, W.; Pan, K.; Gao, J.; Chen, L.; Sun, T.; Zhang, W. Mutations in AtpB and LexA Confer Temperature Tolerance in an Evolved *Synechocystis* Sp. PCC 6803 Strain Resistant to High Temperature and Light. *Plant Physiol. Biochem.* 2026, 235, doi:10.1016/j.plaphy.2026.111384.

57. Hou, G.; Liu, Y.; Liu, W.; Zhang, M.; Ding, H.; Zhou, W. Tolerance Evolution in Cyanobacteria under Chronic Copper and Moxifloxacin Stress: Phenotypic Plasticity and Genomic Fixation. *Algal Res.* 2025, 91, 104327, doi:10.1016/J.ALGAL.2025.104327.

58. Wu, P.; Wei, K.; Hu, T.; Chen, J.; Luan, G.; Sun, L.; Fan, J. Genome-Wide Hypermutation-Engineered *Synechocystis* Sp. PCC 6803 Reveals Membrane-Mediated Triclosan Resistance. *Plant Physiol.* 2026, 200, doi:10.1093/PLPHYS/KIAG121.

59. Hu, L.; Fang, X.; Wen, L.; Zhang, H.; Peng, B.; Li, C. Molecular Insights into the Enhanced Growth of Cyanobacteria by Adaptive Laboratory Evolution in Wastewater Environments. *Algal Res.* 2024, 83, 103724, doi:10.1016/j.algal.2024.103724.

60. Kukil, K.; Englund, E.; Crang, N.; Hudson, E.P.; Lindberg, P. Laboratory Evolution of *Synechocystis* Sp. PCC 6803 for Phenylpropanoid Production. *Metab. Eng.* 2023, 79, 27–37, doi:10.1016/J.YMBEN.2023.06.014.

61. Hosoda, K.; Habuchi, M.; Suzuki, S.; Miyazaki, M.; Takikawa, G.; Sakurai, T.; Kashiwagi, A.; Sueyoshi, M.; Matsumoto, Y.; Kiuchi, A.; et al. Adaptation of a Cyanobacterium to a Biochemically Rich Environment in Experimental Evolution as an Initial Step toward a Chloroplast-Like State. *PLoS One* 2014, 9, e98337, doi:10.1371/JOURNAL.PONE.0098337.

62. Ludwiczak, M.; Dann, M.; Figueroa-Gonzalez, T.; Abdel-Salam, E.M.; Chen, W.; Schwenkert, S.; Lehmann, M.; Zhivkovikj, M.; Noureddine, M.; Linhartová, M.; et al. Photosystem I-Independent Oxygenic Photosynthesis in Cyanobacteria. *Nat. Commun.* 2026, 17, doi:10.1038/S41467-026-74903-2.
